# Giant Isotope Effect in Metal–Organic Frameworks Boosts Unprecedented Photo‐ and Radio‐Luminescence Enhancement

**DOI:** 10.1002/advs.202521054

**Published:** 2025-12-27

**Authors:** Junhao Lu, Dan Zhou, Xianhuan Hao, Hao Lu, Zhongyuan Zhang, Yuhuan Jia, Sen Mei, Ye Huang, Songbai Tang, Zhiyong Peng, Lanhua Chen, Mengjia Yuan, Lixi Chen, Shuaihua Wang, Meiling Feng, Yanlong Wang, Shuao Wang

**Affiliations:** ^1^ State Key Laboratory of Radiation Medicine and Protection School of Radiation Medicine and Protection Collaborative Innovation Center of Radiological Medicine of Jiangsu Higher Education Institutions Soochow University Suzhou P. R. China; ^2^ State Key Laboratory of Structural Chemistry Fujian Institute of Research on the Structure of Matter Chinese Academy of Sciences Fuzhou P. R. China; ^3^ Key Laboratory of Optoelectronic Materials Chemistry and Physics Fujian Institute of Research on the Structure of Matter Chinese Academy of Sciences Fuzhou P. R. China; ^4^ Frontiers Science Center for RareIsotopes Lanzhou University P. R. China; ^5^ New Cornerstone Science Laboratory Suzhou P. R. China

**Keywords:** isotope effect, metal–organic framework, photoluminescence, radioluminescence

## Abstract

Isotope effect (IE) has emerged as a powerful strategy for tailoring the photophysical properties of luminescent systems, yet its application in solid materials remains limited. Herein, we presented a simple and general in situ deuteration strategy to synthesize a series of deuterated metal–organic frameworks (MOFs) with enhanced emission performance. One resulting MOF (EuBTC‐D) exhibits an IE‐triggered comprehensive luminescence enhancement (4.15‐fold longer lifetime and 3.49‐fold higher quantum yield) that surpasses all reported solid‐state deuterated materials. The giant isotope effect is attributed to the drastic suppression of non‐radiative decay in EuBTC‐D, where deuteration effectively freezes the molecule thermal motions of the water‐rich framework and stabilizes the long‐lived triplet excitons. Furthermore, the scope of IE is further extended to radioluminescence, yielding upgraded X‐ray detection sensitivity and imaging resolution. This work establishes isotopic engineering as a versatile and powerful Isotope effect, Metal–organic framework, Photoluminescence, Radioluminescencetool for developing advanced luminescent MOFs and scintillating materials.

## Graphical abstract

Deuterated MOFs in this work exhibited unprecedented giant luminescence isotope effect via O─H oscillator suppression, achieving maximal solid‐state materials emission enhancement. This yields significantly improved X‐ray detection sensitivity and imaging resolution, marking the first integration of MOFs with isotopic luminescence and opening new avenues for radiation detection and X‐ray imaging diagnosis.

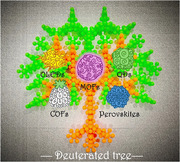

## 1 Introduction

Isotope effect (IE) is the variation in the physical and chemical properties caused by isotopes [1, 2], which share the same atomic number but differ in neutron number. Protium/deuterium is the most extensively studied pair due not only to the relatively low cost of deuterium but also to the most pronounced IE whose magnitude is generally correlated with the relative mass difference between isotopes [3, 4]. The most intriguing feature of IE is its ability to selectively modulate specific properties of a system without altering other properties [5, 6, 7], which is recognized as a non‐invasive strategy for material upgrading. Bearing this benefit, isotopic engineering (primarily utilizing deuterium) has hit a sounding success in pharmaceutical development [8, 9, 10], photoelectric material optimization [11, 12, 13], and reaction mechanism investigation [14, 15]. Despite its enormous potential across various fields, the utility of isotopes remains underexplored, prompting growing research interest in expanding the application scope of IE.

**FIGURE 1:**
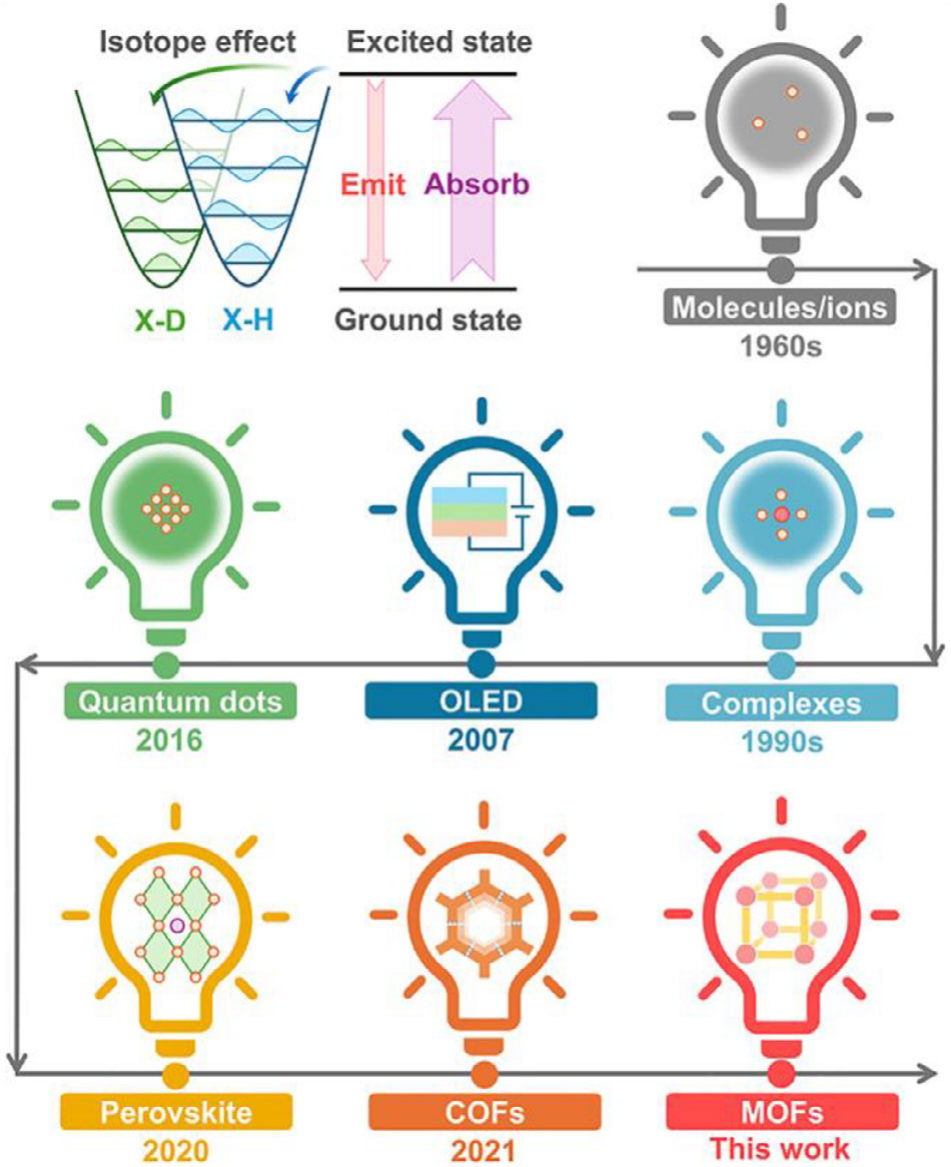
The underlying mechanism of LIE (luminescent isotope effect) and the historical development of LIE.

Luminescent materials are one of the most important constituents that lightens and colorizes the world [16, 17, 18]. The investigation of luminescent isotope effect (LIE) originated in the 1960s [19, 20, 21], when cost‐effective heavy water became commercially available to the chemical research community. Since then, numerous reports documented emission enhancement through either solvent deuteration or luminophore deuteration in solutions, including organic luminescent molecules [22, 23, 24, 25], lanthanide complexes [26, 27, 28, 29, 30, 31], and quantum dots (QDs) [32, 33, 34]. The underlying principle is that heavier isotopes possess lower vibrational energy levels, which thereby weakens the electronic–vibrational (E–V) coupling with solvent molecules and suppresses the dissipation of excited state energy via non‐radiative pathways [35] (Figure 1). Although considerable knowledge of LIE has been accumulated in solution‐state systems, its application in solid‐state luminescent materials had received few attention until recently [6, 7, 11, 12, 25, 26, 36, 37, 38, 39, 40].

The non‐invasive nature of the LIE soon attracted zooming attentions in diverse systems, including organic light emitting diode (OLED) [11, 12, 36, 37, 39, 41, 42, 43, 44, 45, 46, 47, 48], perovskite [49, 50, 51], and covalent‐organic frameworks (COFs) [6, 7]. Since LIE only improves emission intensity while leaving other material properties unchanged, it precludes the possibility of adverse alterations and shortens the R&D cycle [5, 6, 7]. A prominent example is the accelerated lab‐to‐fab transition achieved in deuterated blue OLED [11]. However, the field remains in its infancy, and several key challenges must be addressed: 1) the scope of applicable materials is limited; 2) the enhancement of luminescence is confined to photo‐ and electro‐ luminescence; 3) the deuteration process relies primarily on C─H/D exchange, which often requires harsh reaction conditions. Therefore, there are great interests in expanding the applicability of LIE to enrich the isotopic engineering toolkit.

Luminescent metal–organic frameworks (LMOFs) are cutting‐edge emissive materials with infinite structural diversity, emission tunability and broad applications [52, 53]. Although several reports described the improved luminescence performance of LMOFs in deuterated solvents [54, 55], there is a gap in developing isotopic LMOFs as functional materials. Herein, lanthanide MOFs (Ln‐MOFs) were selected as representative to expand the scope of LIE in three aspects: (1) MOFs were first included into the family of isotopic luminescent materials; (2) radio‐luminescence enhancement by deuteration was verified to enrich the applicable LIE scope; (3) the LIE was achieved by facile O─H deuteration, which was demonstrated to be more efficient than the effect of traditional C─H deuteration.

## 2 Results and Discussion

**FIGURE 2:**
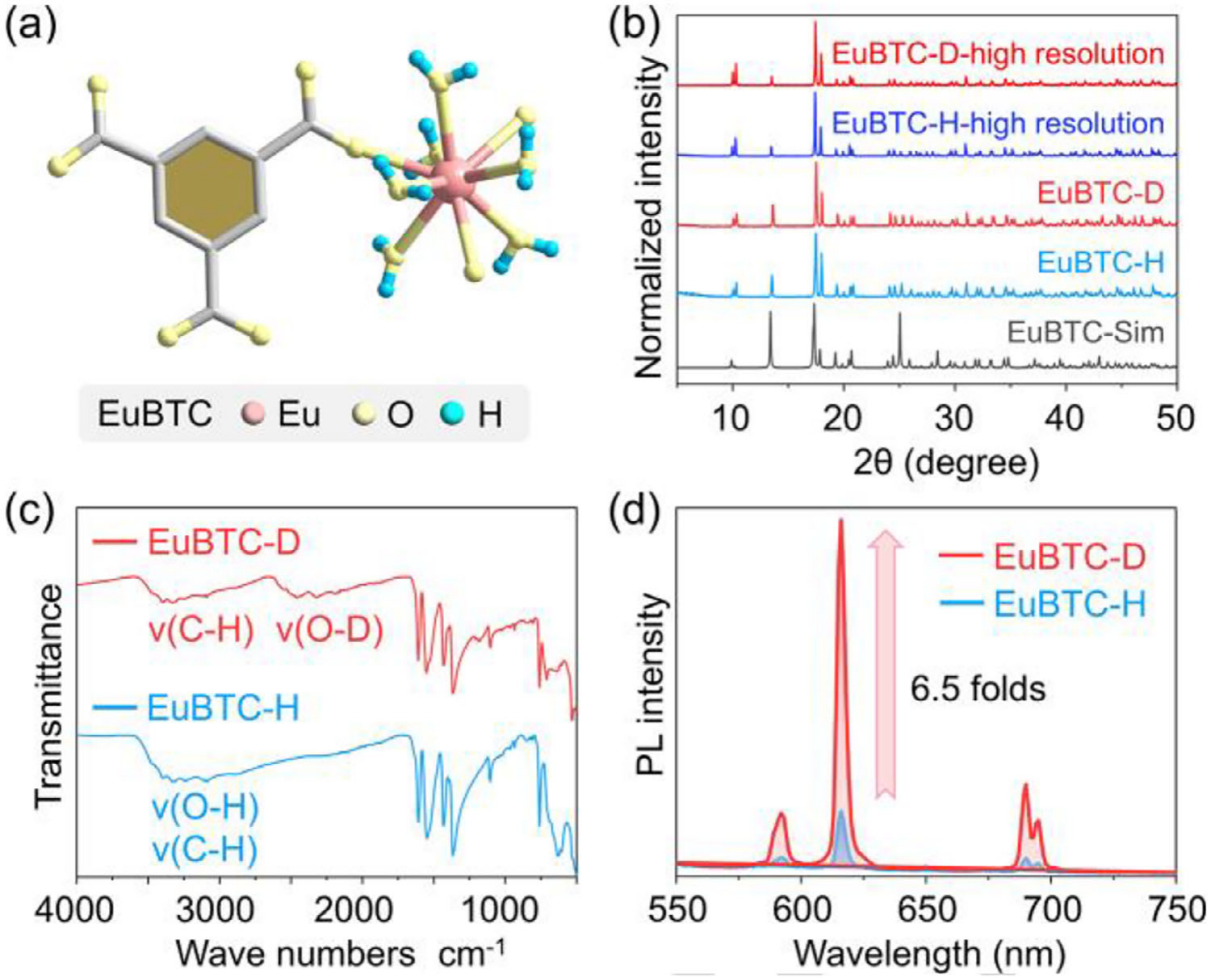
(a) The structure of the model MOF with six water coordination molecules at each Eu site. (b) The conventional and high‐resolution PXRD patterns of the as‐synthesized EuBTC‐H/D and the simulated pattern of the EuBTC. (c) The FT‐IR spectra of the as‐synthesized EuBTC‐H/D. (d) The PL emission spectra of the as‐synthesized EuBTC‐H/D under an excitation (Ex) wavelength at 296 nm.

A lanthanide‐based MOF (noted as EuBTC‐H) [56], prepared by solvothermal reaction of 1,3,5‐benzenetricarboxylic acid (BTC) and Eu(NO_3_)_3_∙(H_2_O)_6_ in DMF/H_2_O mixture, was selected as a model MOF to illustrate how isotope effect could affect the emission property of MOFs. There are six coordinated water molecules on each Eu site and incomplete coordinated carboxyl groups in EuBTC (Figure 2a), both of which serve as potential deuteration sites. The high water content in EuBTC suggests it is more akin to a frozen liquid (Figure 2a; Figure S1), where a network of hydrogen bonds plays an important role in stabilizing the entire framework. The deuterated counterpart (noted as EuBTC‐D) was synthesized through in situ deuteration way by simply replacing the H_2_O with equal volume D_2_O during the solvothermal reaction. Identical to our previous results [5], deuteration does not alter the crystallization thermodynamics and crystallinity, yields crystallographically identical EuBTC‐D with EuBTC‐H (based on conventional and high‐resolution PXRD Figure 2b). Fourier‐transform infrared (FT‐IR) spectra shows that new bands emerge at ∼2400 cm^−1^ for EuBTC‐D (Figure 2c), which can be assigned to O─D stretching vibration. The unchanged characteristic C─H absorption bands at ∼3300 cm^−1^ indicate that the in situ deuteration approach could only deuterate active O─H sites rather than inert C─H sites. This way is advantageous because: (1) the O─H deuteration enjoys much more facile reaction conditions (∼60°C and catalyst‐free for O─H/D exchange in EuBTC vs. >200°C or noble metal catalyzed condition for inert C─H/D exchange [11, 30]) and therefore lower cost; (2) O─H oscillators are closer (directly binding) to the emitting center (Eu) than the remote C─H oscillators, which arouse stronger energy quenching effect [57]; (3) even though at the same distance, O─H oscillators dissipate excited state energy through the E–V coupling more efficiently than C─H oscillators because of its higher vibrational energy and anharmonicity [28, 57]. These features promise an easier approach to achieve more significant LIE.

**FIGURE 3:**
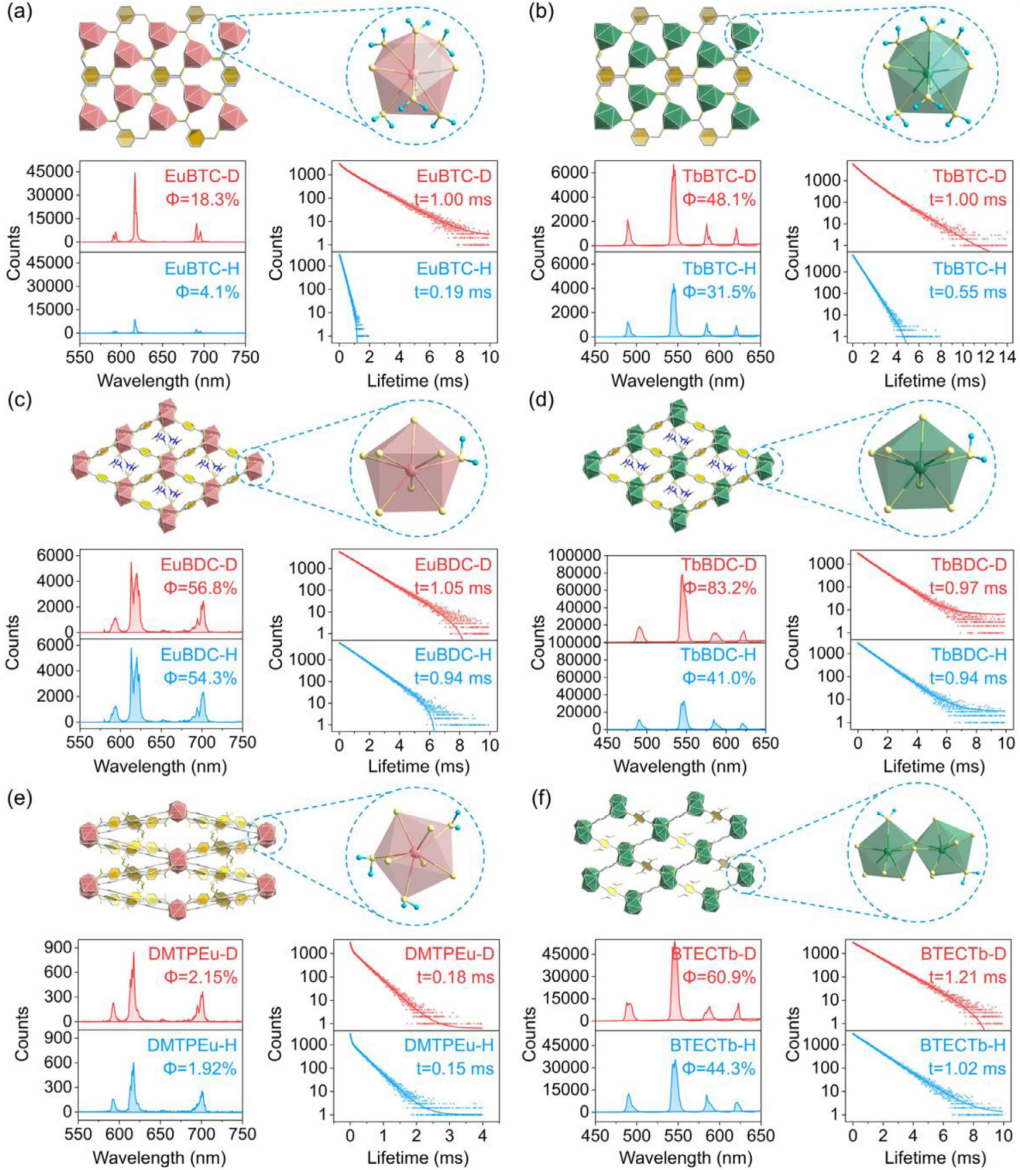
Broad scope of the in situ deuteration strategy on synthesizing outstanding performance Ln‐MOFs. (a–f) Crystal structure of Ln‐MOFs and the coordination information of the Ln ions (top, color legend: Eu, pink; Tb, green; O, light yellow; C, grey; N, deep blue; H, cyan, which were omitted for clarity in crystal structure.), bottom left and bottom right are QY (quantum yield) spectra and LT (lifetime) decay spectra (blue line for H‐system and red line for D‐system), respectively. (a) λ_Ex/Em_ (EuBTC‐H/D) ∼296/616 nm; (b) λ_Ex/Em_ (TbBTC‐H/D) ∼305/544 nm; (c) λ_Ex/Em_ (EuBDC‐H/D) ∼298/616 nm; (d) λ_Ex/Em_ (TbBDC‐H/D) ∼298/544 nm; (e) λ_Ex/Em_ (DMTPEu‐H/D) ∼366/616 nm; (f) λ_Ex/Em_ (BTECTb‐H/D) ∼313/544 nm.

As expected, EuBTC‐D exhibits upgraded photoluminescence (PL) than EuBTC‐H, resulting in 650%, 515%, and 449% increasing in PL intensity, lifetime (LT) and quantum yield (QY), respectively (Figures 2d and 3a; Figures S2–S4). Such gaint IE is rare in solid‐state luminescent materials, which will be discussed later. As a comparison to in situ deuteration strategy, a post‐deuteration experiment in which the as‐synthesized EuBTC‐H powder was soaked in D_2_O for O─H/D exchange was also carried out (noted as EuBTC‐D‐post). EuBTC‐D‐post shows lower deuteration degree (the deuterium abundance of EuBTC‐H, EuBTC‐D‐post and EuBTC‐D are 0.0163%, 0.1552%, and 16.27%, respectively, Table S1) and no significant enhancement in photoluminescence properties compared to EuBTC‐H while its structure remained intact based on the high‐resolution PXRD results (Figures S5–S8), highlighting the advantage of in situ deuteration for pursuing highly deuterated materials that thoroughly release the potential of LIE.

Considering the cost of D_2_O, control experiments in which the H_2_O in the EuBTC synthesis process was 20%, 60%, and 80% replaced by D_2_O were performed to ascertain whether the observed enhancement of photoluminescence is directly attributable to the D_2_O medium. As shown in Figures S9–S13, it demonstrated a progressive increase in both fluorescence LT and QY with increasing heavy water content. The in situ deuteration strategy is also effective for Ln‐MOF containing N─H sites, to avoid the interference from the O─H sites, a Ln‐MOF (EuNH_2_BDC) that does not contain ─OH sites in its structure were chosen to verify the effectiveness of deuteration strategy [58]. As shown in Figures S14–S18, the LT and QY were slightly promoted from 0.328 to 0.340 ms and 3.91% to 4.42%, respectively, the enhancement is much smaller than that of OH deuteration which could be contributed to the fact that: 1) ─NH is more difficult to be deuterated than ─OH [59, 60], resulted lower deuteration degree; (2) the ─NH groups are located on the benzene ring that are not directly binding to the emitting centers, which possesses a relatively weaker energy quenching effect [57].

**FIGURE 4:**
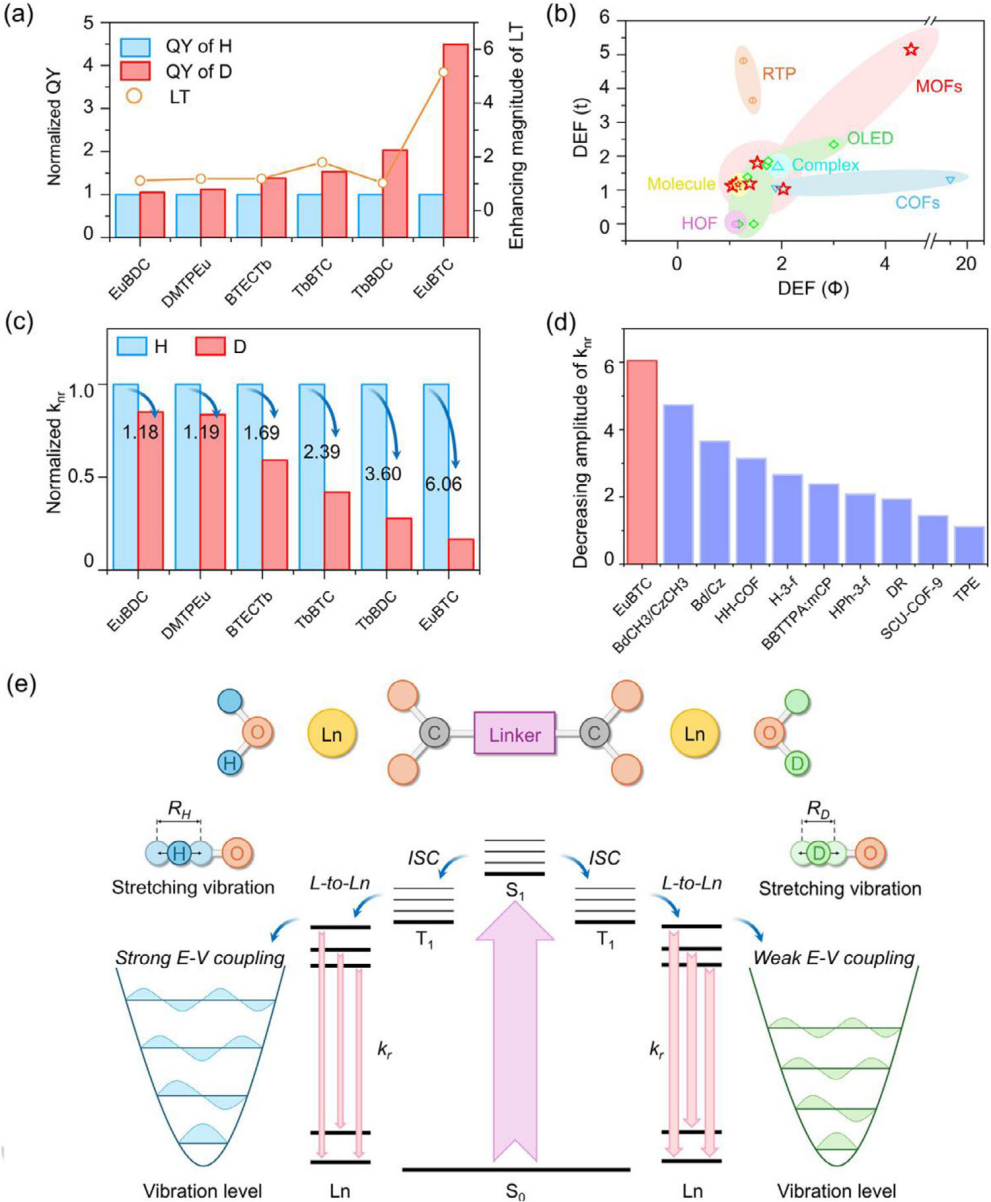
(a) Enhancing magnitude in LT and QY after deuteration (the data were normalizaed by the standard of hydrogenated MOFs). (b) Comparison between deuterated MOFs and other materials in terms of DEF(t) and DEF(Φ), DEF means deuteration effect factors. (c) Decreasing magnitude of k_nr_ in the prepared Ln‐MOFs after deuteration (the data were normalizaed by the standard of hydrogenated MOFs). (d) Comparison between deuterated MOFs and other materials in terms of decreasing magnitude of k_nr_. (e) Schematic illustration of the transition pathways in the photoluminescence process of EuBTC‐H/D.could easily reach over one order of magnitude, Table S4), so in solids the thermal motion‐caused quenching is relatively weaker and the LIE, which freezes the bond vibration, is less pronounced.

Owing to the large ionic radii and high coordination numbers characteristic of Ln^3+^ ions, the organic ligands in Ln‐MOFs are often insufficient to satisfy their full coordination environment [61, 62]. This results in vacant coordination sites, which are typically occupied by water molecules. To this regard, Ln‐MOFs present themselves as promising candidates for in situ deuteration—a general strategy for the preparation of deuterated MOFs. Simply substituting H_2_O with D_2_O during synthesis yields structurally identical deuterated Ln‐MOFs. As anticipated, the deuterated Ln‐MOFs synthesized from different Ln ions and organic ligands generally exhibit enhanced PL intensity, higher QY, and prolonged LT compared to their hydrogenated analogues (Figure 3; Figures S19–S43). As summarized in Figure 4a and Table S2, the extent of improvement resulting from deuteration varies: luminescence lifetime enhancements range from 103% (TbBDC) to 515% (EuBTC), quantum yield enhancements span from 105% (EuBDC) to 449% (EuBTC). These results confirm the general applicability of the LIE strategy in Ln‐MOFs. Notably, among all deuterated solid‐state materials, the present systems achieve the highest improvement in LT and the second highest in QY (Table S3). We attribute the observed variation in enhancement to differences in crystal structure and the quantity of coordinated water molecules: In structurally rigid frameworks (e.g., Eu/TbBTC‐H/D, Eu/TbBDC‐H/D, and BTECTb‐H/D), Ln‐MOFs with a greater number of coordinated water molecules tend to exhibit more pronounced LIE. In particular, for Eu/TbBTC‐H/D, the presence of a large number (six) of coordinated water molecules and exposed ─COOH groups contributes substantially to non‐radiative decay pathways. In such case, deuteration leads to maximum enhancement relative to MOFs containing only one coordinated water molecule (Figures 3c,d,f and 4c; Table S2). In contrast, for the structurally more flexible DMTPEu‐H/D, non‐radiative transitions are dominated by framework fluctuations or deformations rather than bond vibrations. Therefore, despite having the second‐highest number (three) of coordinated water molecules, deuteration in DMTPEu leads to only negligible enhancement (Figures 3e and 4c; Table S2). It should be noted that LIE enhancement varies even when the coordination environment and cyrstal structure are identical, such as EuBTC‐H/D exhibits much more pronounced IE enhancement, which may deprived from the vibrational coupling difference between the O─H/D oscillators and the Eu^3+^/Tb^3+^ ions. As shown in Figure S44, the Eu^3+^ has an energy gap of ∼16200 cm^−1^ (616 nm) between its emissive D_0_ level and the lower ^7^D_2_ state, this gap is strongly resonant with the fifth harmonic overtone of the O─H stretching vibration for the H‐system and weakly resonant with the sixth harmonic overtone of the O─D stretching vibration for the D‐system, which is manifested as the emission spectral overlap between Eu^3+^ and the O─H/D overtones absorption bands. Hence the deuteration strategy could weaken the nonradiative decay pathways and significantly enhance the luminescence ability. While the Tb^3+^ (544 nm) has an energy gap of ∼18380 cm^−1^ between its emissive ^5^D_4_ level and the lower ^7^F_5_ state, this gap is resonant with the sixth harmonic overtone of the O─H stretching vibration and the seventh harmonic overtone of the O─D stretching vibration, the emission spectral overlap between Tb^3+^ and the O─H/D overtones absorption bands are smaller than that of Eu^3+^, which may contributed to the more pronounced IE enhancement in EuBTC‐H/D.

A comparison with reported solid‐state deuterated materials (including OLEDs, COFs, HOFs) further highlights the superiority of deuterated MOFs (Figure 4b). For clarity, the Deuteration Effect Factors (DEF) in LT and QY were defined as DEF(t) = LT(D)/LT(H) and DEF(Φ) = QY(D)/QY(H), respectively. Most materials located in the area of DEF(t)<2 and DEF(Φ)<2. This is reasonable because: (1) IE is commonly weak in most cases since the vibrational frequency of X─D is roughly ∼70% that of X─H (calculation details in statistical analysis); (2) the structures of solid‐state materials are much more rigid than those in solutions [63, 64] (where DEF

Only limited materials exhibit a giant LIE with a DEF(t) or DEF(Φ) exceeding 3 (even in MOF system, EuBTC‐H/D, Figure 4b). Liu reported significant LIE in room temperature phosphorescence (RTP) systems with DEF(t) = 4.73 and DEF(Φ) = 1.26 [38]. The LIE was found to significantly prolong the triplet state excitons and increase the intersystem crossing (ISC) rate of the deuterated RTP. We previously reported a deuterated COF (SCU‐COF‐9) that show an astoundingly high DEF(Φ) = 19 (while DEF(t) is only 1.32) [7]. Interestingly, the IE on the non‐radiative decay rate (k_nr_) was at average level (k_nr_(D)/k_nr_(H) = 0.69), whereas its effect on the radiative decay rate (k_r_) was substantially enhanced (k_r_(D)/k_r_(H) = 16.8). This abnormal result likely arises not solely from the LIE but from a combination of factors (e.g., IE on crystallinity [40, 49]). The EuBTC‐H/D presented here represented the only case with significant enhancement on both luminescence lifetime and quantum yield (DEF(t) = 5.15, DEF(Φ) = 4.49), which outcompetes all the reported solid‐state deuterated materials (Tables S2 and S3). Since the triplet state excitons are particularly vulnerable to non‐radiative deactivations [65, 66], the simultaneous improvement of the exciton lifetime and radiative efficiency constitutes a trade‐off problem in Ln‐MOFs since its photoluminescence mechanism includes S_0_→S_n_→T_n_→Eu pathway (Figure 4e). The current result obviously offers a feasible isotope tool to solve this trade‐off issue.

The k_r_ and k_nr_ were calculated according to k_total_ = 1/LT, k_r_ = QY/LT, k_nr_ = k_total—_k_r_ equations to deepen the understanding of how LIE works in MOFs. As summarized in Table S2, deuteration significantly reduces the k_nr_ and slight changes the k_r_, leading to growing proportion of k_r_ in k_total_ in deuterated MOFs than the original ones (Figure S45). Aligning to the above results, the most significant reduction extent in k_nr_ was found in EuBTC‐H/D (Figure 4c), which is also the largest reduction among all reported deuterated solid materials (Figure 4d; Tables S2 and S3). All the above evidence indicated that EuBTC was a unique case that exhibits unprecedentedly giant LIE. The underlying mechanism may derive from three aspects: (1) Compared to the rigid and dense structure of reported deuterated materials, EuBTC possessing high water content is relatively more flexible due to the rich hydrogen bonds, which are idea pathway for non‐radiative transition. Deuteration could frozen these oscillating weak bonds; (2) The emitting center in other deuterated materials is largely organic entity that involves C─H/D deuteration, while the Eu in EuBTC is surrounded by water, which contains O─H oscillators as stronger quencher. Deuteration at O─H sites works better than at C─H sites that mentioned above (this was also verified by experiments using MOFs with deuterated ligand. Two MOFs (Tbd‐BDC‐H/D) synthesized using the 1,4‐Benzene‐2,3,5,6‐d4‐dicarboxylicacid (d‐BDC) were compared to the Tb‐BDC‐H/D (Table S5; Figures S46–S50). When a Ln MOF is deuterated on both O─H and C─H sites, the PL enhancement can be additive and O─H deuteration works better than C─H deuteration); (3) Most deuterated materials are fluorescent that involves short‐lived singlet state excitons while EuBTC is phosphorescence whose long‐lived triplet state excitons are sensitive to thermal motion‐related energy dissipation pathway, rendering its deuteration more effective in decoupling the undesired electronic‐vibrational energy transfer.

**FIGURE 5:**
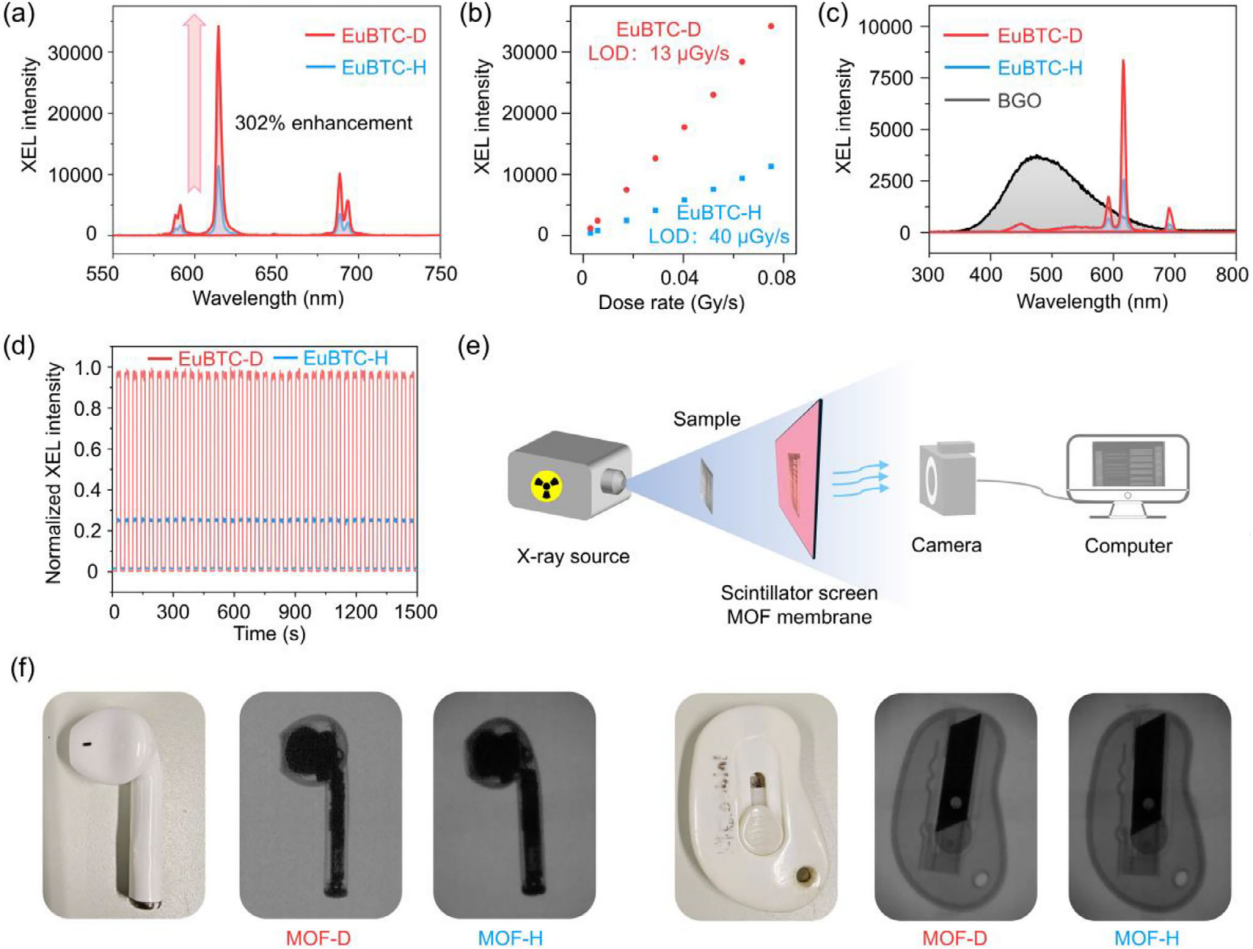
Comparison of XEL detection and imaging performance of EuBTC‐H/D. (a) The XEL spectra of EuBTC‐H/D under an X‐ray dose rate of 0.075 Gy/s. (b) The XEL intensity vs dose rate ranging from 0.003 to 0.075 Gy/s. (c) The XEL spectra of EuBTC‐H/D and BGO was collected with a specially calibrated X‐ray source under a certain dose rate (0.042 Gy/s) in which the X‐ray energy photon yields of BGO is 8000 photons/MeV. (d) Reusability of the EuBTC‐H/D. Each cycle received a dose rate of 0.0442 Gy/s, and a total of 37 on‐off cycles were repeated (40 s/cycle). (e) A schematic illustration of the X‐ray imaging device. (f) The images of a headset and a knife (physical objects). And their X‐ray images conducted with the flexible films based on powders of EuBTC‐H/D.

Most of the aforementioned deuterated materials are based on photoluminescence or electroluminescence aiming at improving the performance of displaying, lighting, or sensing [12, 24, 40, 41, 48]. In an effort to broaden the application scope of the LIE strategy, we explored its potential in radioluminescence by evaluating the X‐ray excited luminescence (XEL) performance of EuBTC‐H and its deuterated analogue, EuBTC‐D. To date, only one deuterated semiconductor‐type perovskite has been reported for X‐ray detection based on its electroconductibility rather than luminescence [50]. As shown in Figure 5a, consistent with the enhancements observed in PL, LT, and QY, EuBTC‐D exhibited a markedly higher XEL intensity than EuBTC‐H under the same dose rate (0.075 Gy/s), surpassing the latter by 302%. Both EuBTC‐H and EuBTC‐D displayed excellent linearity in XEL intensity over a dose range of 0.003–0.075 Gy/s (R^2^ = 0.999, Figure 5b; Figure S51). Based on the slope of the linear fit between dose rate and XEL intensity, the limit of detection (LOD) for X‐rays was calculated to be 39.56 µGy/s for EuBTC‐H and 13.05 µGy/s for EuBTC‐D, corresponding to a 303% improvement in sensitivity after deuteration. We furtherly evaluated the X‐ray energy photon yields, irradiation stability, and reusability of both materials. Under fixed conditions (100 kV, 50 µA, 0.042 Gy/s), the reference sample BGO (Bi_4_Ge_3_O_12_) exhibited an X‐ray energy photon yield of 8000 photons/MeV. By integrating the XEL spectra, the yields for EuBTC‐H and EuBTC‐D were estimated as 1114.8 and 2459 photons/MeV, respectively (Figure 5c). After exposure to a cumulative γ‐ray dose of 50 kGy (1 kGy/h), PXRD confirmed that the crystal structure of both materials remained intact, indicating that deuteration does not compromise irradiation stability, and the O─D stretching vibration in the FT‐IR spectrum confirmed that the coordinated D_2_O molecules still anchored to the Eu^3+^ ions. (Figures S52 and S53). It should be noted that the XEL performance after γ‐ray exposure decreased 45% and 33% for EuBTC‐H/D respectively which may be caused by local defects and changes in the electronic structure by radiation (Figure S54). These factors do not affect the long‐range order, but are sufficient to quench the excited‐state energy [67]. Reusability tests of EuBTC‐H/D over 37 on–off cycles revealed no significant degradation in XEL performance, furtherly confirming that deuteration preserves material robustness under repeated irradiation (Figure 5d). In addition, the EuBTC‐H/D remains relatively stable XEL performance within 90 Gy X‐ray exposure (14.3% and 13.7% drop in intensity for EuBTC‐H and EuBTC‐D respectively), exhibited the scintillating stability under continuous radiation exposure (Figure S55). The lower decrease for EuBTC‐D may derive from the stronger bond strength of O─D over O─H so that better radiation stability was witnessed for deuterated MOFs. Similar results were also reported in OLEDs where deuterated ones show better light irradiation stability over original ones [11, 48]. Given the superior X‐ray detection performance of EuBTC‐D, we investigated its potential in X‐ray imaging by fabricating flexible composite films using EuBTC‐H or EuBTC‐D

Powders embedded in a transparent polydimethylsiloxane (PDMS) matrix, as shown in Figure S56, the membrane fabricated with EuBTC‐H/D powders are identical in appearance. Scanning electron microscope (SEM) images and energy‐dispersive spectroscopy spectroscopy (EDS) analysis have shown the even distribution of MOF particles and elements on the PET matrix membrane. No obvious MOF agglomeration phenomenon was spotted. Thus, luminescence variation in EuBTC‐H/D was assigned to deuteration rather than particle agglomeration. As illustrated in Figure 5e, the imaging setup involved placing the test object between the X‐ray source and the scintillator film, with images captured by a digital camera on the opposite side. The EuBTC‐D‐based film produced visibly sharper and more intense images than its hydrogenated counterpart, attributable to its enhanced radioluminescence, based on the modulation transfer function (MTF) data, the the imaging resolutions of the two films are 2.27 (H) and 3.97 (D) respectively. (Figure 5f; Figures S57 and S58). Moreover, imaging tests conducted at different exposure times revealed that the EuBTC‐D film achieved high‐quality images even under shorter exposures, suggesting a potential route to reducing ionizing radiation doses in medical diagnostics (Figure S59). These results demonstrate a general and straightforward deuteration strategy for enhancing the scintillating and imaging performance without altering fundamental optical properties, irradiation stability, or reusability. This approach can be readily extended to other metal–organic framework scintillators and their derived flexible films.

## 3 Conclusion

This work pioneers the incorporation of MOFs into the family of isotope effect luminescent materials via a universal in situ O─H deuteration strategy. The deuterated EuBTC‐D MOF achieves a record‐breaking enhancement in solid‐state luminescence, exhibiting a 515% increase in lifetime and a 449% improvement in quantum yield, while its radioluminescence intensity and X‐ray detection sensitivity are also boosted by over 300%. This unprecedented isotope effect originates from the direct coordination of high‐energy O─H oscillators to the Eu^3+^ center, enabling more effective suppression of non‐radiative decay compared to conventional C─H deuteration. The demonstrated radioluminescence enhancement opens new avenues for developing high‐performance scintillators, significantly advancing isotopic engineering applications in medical imaging, radiation detection, and photonic devices.

## 4 Experimental Section

### 4.1 Materials

All chemicals and solvent, were purchased from commercial chemical reagent suppliers and used directly without further purification. Ultrapure water (H_2_O) was accessed from a Milli‐Q purification system (Merck KGaA).

### 4.2 Synthetic Methods

Synthesis of EuBTC‐H/D [56] (0/100%D_2_O@EuBTC): 45 mg of 1,3,5‐benzenetricarboxylic (BTC) was ultrasonically dissolved in 13.5 mL N,N‐dimethylformamide (DMF), then added 9 mL H_2_O. 45 mg of Eu(NO_3_)_3_·6(H_2_O) was ultrasonically dissolved in 9 mL H_2_O, then added 13.5 mL DMF. Both the two solutions were mixed and filled into three 20 mL glass vials, then the vials were sealed and placed in a preheated oven at 60°C for 168 h. EuBTC‐D was synthesized using equal volume of D_2_O to replace the H_2_O in the same procedure.

Synthesis of 20/60/80%D_2_O@EuBTC [56] the synthesis process is identical to the EuBTC‐H, unless the H_2_O was partially (20/60/80%) replaced by D_2_O.

Synthesis of 10/30%Gd@EuBTC‐D [56] the synthesis process is identical to the EuBTC‐D, unless the Eu(NO_3_)_3_·6(H_2_O) was partially (10/30% in molar) replaced by Gd(NO_3_)_3_·6(H_2_O).

Synthesis of TbBTC‐H/D [56] 45 mg of 1,3,5‐benzenetricarboxylic (BTC) was ultrasonically dissolved in 13.5 mL DMF, then added 9 mL H_2_O. 46 mg of Tb(NO_3_)_3_·6(H_2_O) was ultrasonically dissolved in 9 mL H_2_O, then added 13.5 mL DMF. Both the two solutions were mixed and filled into three 20 mL glass vials, then the vials were sealed and placed in a preheated oven at 60°C for 168 h. EuBTC‐D was synthesized using equal volume of D_2_O to replace the H_2_O in the same procedure.

Synthesis of EuBDC‐H/D: 104 mg EuCl_3_·6(H_2_O) was dissolved in 2 mL H_2_O. 66 mg 1,4‐dicarboxybenzene (H_2_BDC) was ultrasonically dissolved in 10 mL DMF. Then the two filtrates were mixed in a 20 mL clamp‐cap glass vial. Then the vial was sealed and placed in a preheated oven at 90°C for 48 h. Transparent crystals (EuBDC‐H) were formed on the wall and bottom of the glass vial after naturally cooling down to room temperature. EuBDC‐D crystals were synthesized using equal volume of D_2_O to replace the H_2_O in the same procedure.

Synthesis of TbBDC‐H/D [68] 107 mg of TbCl_3_·6(H_2_O) was dissolved in 2 mL H_2_O. 66 mg H_2_BDC was ultrasonically dissolved in 10 mL DMF. Then the two filtrates were mixed in a 20 mL clamp‐cap glass vial. Then the vial was sealed and placed in a preheated oven at 90°C for 48 h. Transparent crystals (TbBDC‐H) were formed on the wall and bottom of the glass vial after cooling down to room temperature. TbBDC‐D crystals were synthesized using equal volume of D_2_O to replace the H_2_O in the same procedure.

Synthesis of Tbd‐BDC‐H/D [68] the synthesis process is identical to the TbBDC‐H/D, unless the H_2_BDC was replaced by 1,4‐benzene‐2,3,5,6‐d4‐dicarboxylicacid (d‐BDC).

Synthesis of DMTPEu‐H/D [69] 20 mg of 2′,5′‐ dimethoxytriphenyl‐4,4″‐dicarboxylic acid (DMTP) was ultrasonically dissolved in 6 mL DMF. 25 mg of Eu(NO_3_)_3_·6(H_2_O) was ultrasonically dissolved in 4 mL H_2_O. Both the two solutions were mixed and 20 µL lactic acid was added, the mixture was filled into two Teflon lined stainless vessels, then the vials were sealed and placed in a preheated oven at 140°C for 72 h. DMTPEu‐D was synthesized using equal volume of D_2_O to replace the H_2_O in the same procedure.

Synthesis of BTECTb‐H/D [70] 114.4 mg of 1,2,4,5‐benzenetetracarboxylic acid (BTEC) and 168 mg of TbCl_3_·6(H_2_O) were ultrasonically dissolved in 23 mL H_2_O, then the mixture was filled into two Teflon lined stainless vessels, then the vials were sealed and placed in a preheated oven at 130°C for 72 h. BTECTb‐D was synthesized using equal volume of D_2_O to replace the H_2_O in the same procedure.

Synthesis of EuNH_2_BDC‐H/D [58] 30 mg of 2‐aminoterephthalic acid (NH_2_BDC) and 50 mg of Tb(NO_3_)_3_·6(H_2_O) were ultrasonically dissolved in 0.2 mL H_2_O and 4.8 mL DMF, then the mixture was filled into two Teflon lined stainless vessels, Then the vial was sealed and placed in a preheated oven at 120°C for 48 h. EuNH_2_BDC‐D was synthesized using equal volume of D_2_O to replace the H_2_O in the same procedure.

Synthesis of YbNDC‐H/D [71] 42 mg of Naphthalene‐2,6‐dicarboxylic acid (NDC) and 10 mg of 4,4‐Bipyridine were ultrasonically dissolved in 4 mL DMF. 47 mg Yb(NO_3_)_3_·6(H_2_O) was dissolved in 2 mL H_2_O.Then the mixture was filled into two Teflon lined stainless vessels, Then the vial was sealed and placed in a preheated oven at 60°C for 96 h. YbNDC‐D was synthesized using equal volume of D_2_O to replace the H_2_O in the same procedure.

Fabrication of flexible scintillator film of EuBTC‐H/D: First, the MOF powder was thoroughly grounded and sieved. Then, it was thoroughly and evenly mixed with the commercially purchased curing agent (epoxy resin). The mixture was evenly applied onto the PET (polyethylene terephthalate) substrate, and dried and cured in the oven at 70°C, resulting in a flexible membrane made of the MOF scintillator.

### 4.3 Instruments

The PXRD experiments were performed on a Bruker D8 Advance Diffractometer with Cu Kα radiation (λ = 1.54056 Å), the data were collected from 5 to 50° with a step of 0.02° (0.002° for high resolution PXRD). The SEM‐mapping experiments were performed on a Quanta 400 FEG Field emission environmental scanning electron microscope. The photoluminescence (PL) emission and excitation spectra were collected using an Edinburgh FLS980 steady‐state fluorimeter. Photoluminescence quantum (QY) yields measurements were performed on FLS980 and Integrating Sphere Assembly F‐M01 by direct integrating sphere mechanism. The Fourier Transform infrared spectra (FT‐IR) were measured on a Thermo Nicolet 6700 spectrometer. The isotope abundance test was conducted on a Gas Chromatography/Elemental Analysis/Water Balance Stable Isotope Mass Spectrometer. The X‐ray excited luminescence (XEL) spectra were collected X‐RAD SmART system equipped with W Kα radiation source and a NOVA spectrometer (ideaoptics, China) with an adjustable dose rate controlled by changing the current of X‐ray source. The X‐ray images were captured using a digital camera for photography (Canon EOS 5D Mark IV with EF 24–70 mm f/2.8 L II USM lens).

### 4.4 Statistical Analysis

The raw data of all the experiments mentioned above were all processed by the Origin 2021 software (including the fitting results of the lifetime and the LOD). The modulation transfer function (MTF) data was calculated by the MatLab software based on the X‐ray image of the line pair card. In order to facilitate the comparison of the H/D system, the data of PXRD and FT‐IR were normalized. The limit of detection (LOD) was calculated by LOD = 3σ/slope [72] where σ is the standard deviation calculated by repeated tests of background signals for eleven times, and k is the slope of linear fitting curve. It should be noted that MOF powders (25 mg) were used for XEL testing instead of bulk crystals to exclude the influence of crystal morphology of different doping samples on the XEL performance. For a diatomic molecule A─B, its vibration frequency (wavenumber) ν̃ is given by the following formula, in which c represents the speed of light; k is the force constant, representing the strength of the chemical bond. For the same chemical bond (such as C─H and C─D), the value of k is almost the same; μ represents the reduced mass (Take C─H/D as an instance, µ_C‐H_ ≈ 0.923u, µ_C‐H_ ≈ 11.714u, ν̃_C‐D_/ν̃_C‐H_ ≈ 0.734 (in the same way, ν̃_N‐D_/ν̃_N‐H_ ≈ 0.730, ν̃_O‐D_/ν̃_O‐H_ ≈ 0.727).

(1) $$\tilde{\nu }\;=\;\frac{1}{2\pi c}\sqrt{\frac{k}{\mu }}$$

(2) $$\frac{{\tilde{\nu }}_{X-D}}{{\tilde{\nu }}_{X-H}}=\sqrt{\frac{\mu_{X-H}}{{\tilde{\mu }}_{X-D}}}$$

(3) $$\mu =\frac{m_{A}m_{B}}{m_{A}+m_{B}}$$

## Conflicts of Interest

The authors declare no conflicts of interest.

## Supporting information




**Supporting File**: advs73568‐sup‐0001‐SuppMat.docx.

## Data Availability

All data can be found in Supporting Information.
