# Supplementary material for: Giant Isotope Effect in Metal–Organic Frameworks Boosts Unprecedented Photo‐ and Radio‐Luminescence Enhancement
Source: Adv Sci (Weinh). 2025 Dec 27;13(14):e21054. doi: 10.1002/advs.202521054 (PMC12970278; doi:10.1002/advs.202521054)
Supplement: Supplementary file 1 — Supporting File: advs73568‐sup‐0001‐SuppMat.docx. [file ADVS-13-e21054-s001.docx]

**Giant Isotope Effect in Metal-Organic Frameworks Boosts Unprecedented Photo- and Radio-luminescence Enhancement**

Junhao Lu^1#^, Dan Zhou^1,4#^, Xianhuan Hao^1^, Hao Lu^3^, Zhongyuan Zhang^3^, Yuhuan Jia^1^, Sen Mei^1^, Ye Huang^1^, Songbai Tang^1^, Zhiyong Peng^1^, Lanhua Chen^1^, Mengjia Yuan^1^, Lixi Chen^1,^ *, Shuaihua Wang^3^, Meiling Feng^2,^*, Yanlong Wang^1, 2^*, Shuao Wang^1,5^*

[#] These authors contributed equally.

[1] J. Lu, Dr. D. Zhou, X. Hao, Y. Jia, S. Mei, Y. Huang, Dr. S. Tang, Dr. Z. Peng, Dr. L. Chen, Dr. M. Yuan, Dr. L. Chen, Prof. Y. Wang, and Prof. S. Wang
State Key Laboratory of Radiation Medicine and Protection, School of Radiation Medicine and Protection, Collaborative Innovation Center of Radiological Medicine of Jiangsu Higher Education Institutions,
Soochow University, Suzhou 215123, China

E-mail: [shuaowang@suda.edu.cn](mailto:shuaowang@suda.edu.cn); [ylwang@suda.edu.cn](mailto:ylwang@suda.edu.cn); [lxchen@suda.edu.cn](mailto:lxchen@suda.edu.cn)

[2] Prof. M. Feng, Prof. Y. Wang

State Key Laboratory of Structural Chemistry, Fujian Institute of Research on the Structure of Matter, Chinese Academy of Sciences,
Fuzhou 350002, P. R. China

E-mail: [ylwang@suda.edu.cn](mailto:ylwang@suda.edu.cn); [fml@fjirsm.ac.cn](mailto:fml@fjirsm.edu.cn)

[3] H. Lu, Z. Zhang, Prof. S. Wang

Key Laboratory of Optoelectronic Materials Chemistry and Physics, Fujian Institute of Research on the Structure of Matter, Chinese Academy of Sciences, Fuzhou 350002, P. R. China

E-mail: [shwang@fjirsm.ac.cn](mailto:shwang@fjirsm.ac.cn)

[4] Dr. D. Zhou

Frontiers Science Center for RareIsotopes, Lanzhou University 730000, P. R. China

[5] Prof. S. Wang

New Cornerstone Science Laboratory, Suzhou 215123, China

Table of Contents

**Section S1. Supplementary figures**

**Section S2. Supplementary tables**

**Section S3. References**

**Section S1. Supplementary figures**

**
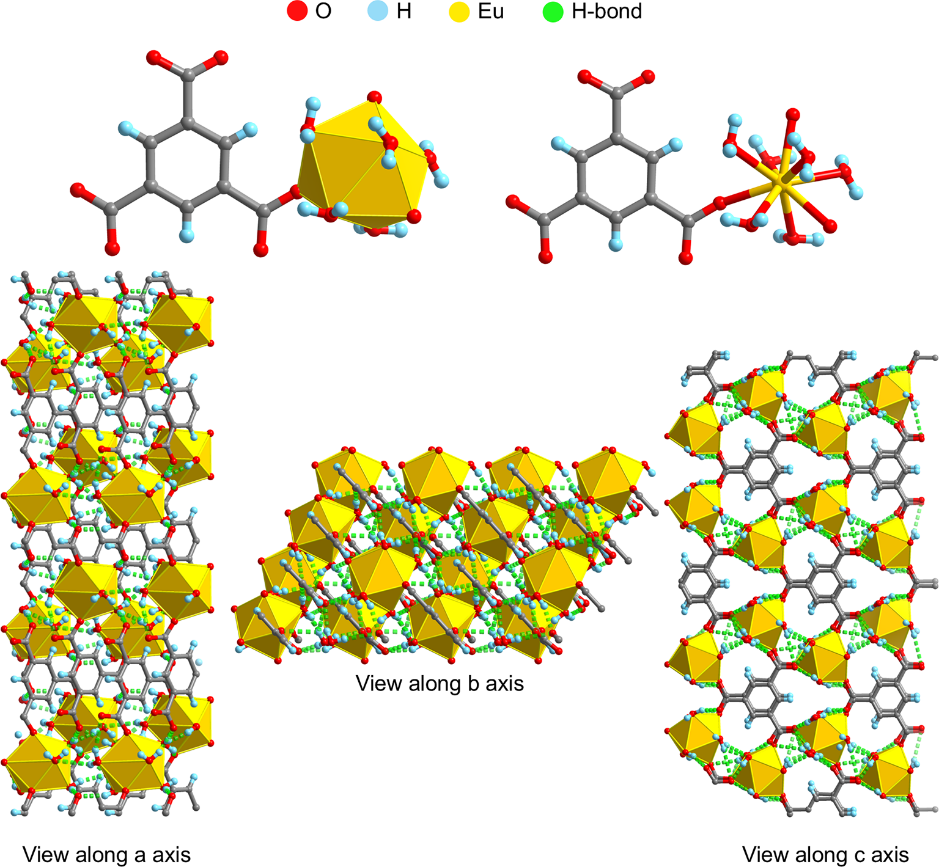
**

**Figure S1.** The structure information of EuBTC.

**
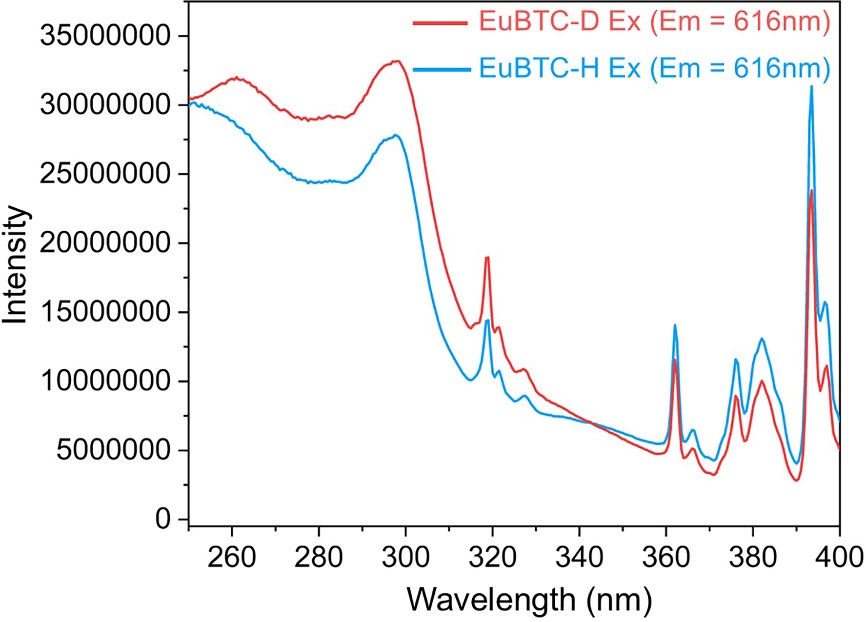
**

**Figure S2.** The photoluminescence excitation spectra of EuBTC-H/D under the emission (Em) wavelength at 616 nm.


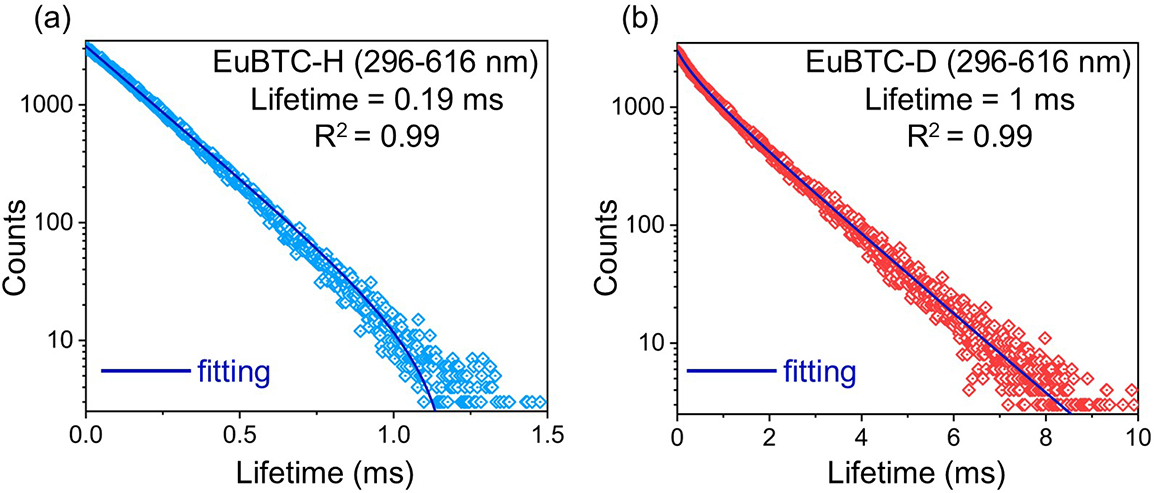


**Figure S3.** Fitting of the lifetime of EuBTC-H/D based on PL data (Ex/Em = 296/616 nm).


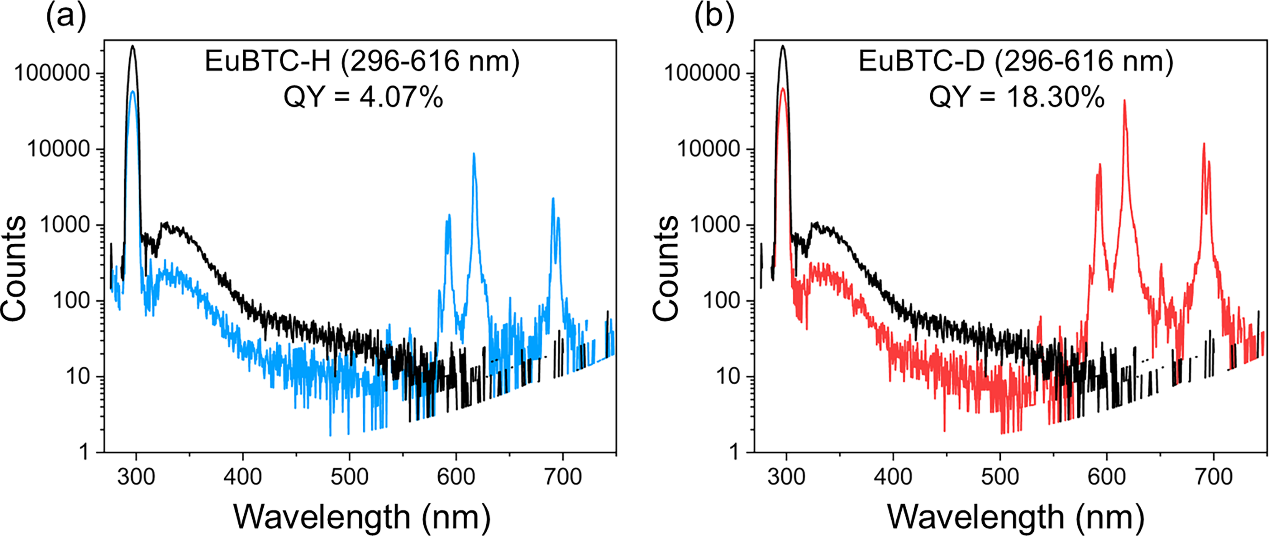


**Figure S4.** Quantum yield of EuBTC-H/D based on PL data (Ex/Em = 296/616 nm, blue line for H, red line for D, black line for background).


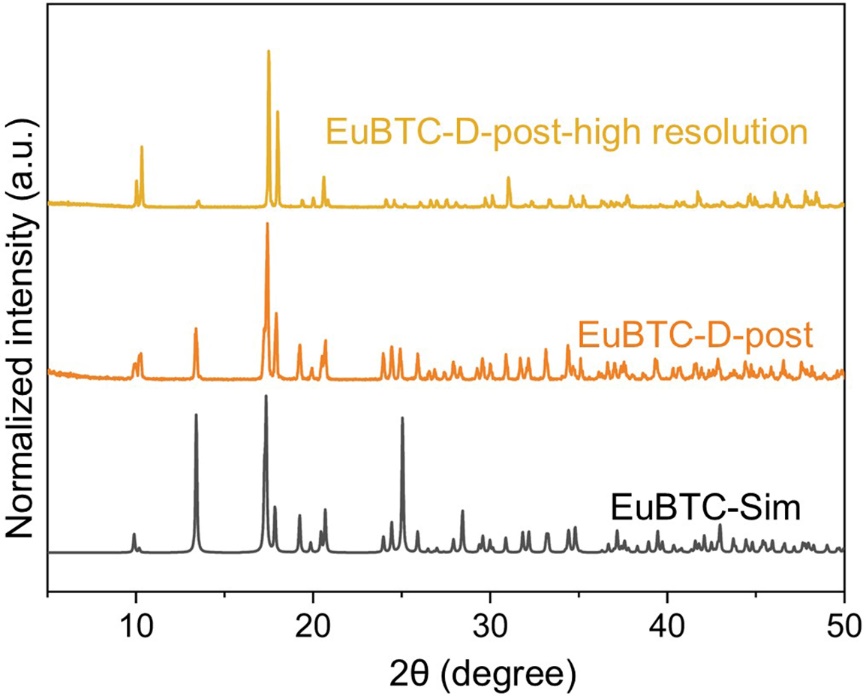


**Figure S5.** Comparison of PXRD patterns between EuBTC-D-post (regular-resolution PXRD), EuBTC-D-post (high-resolution PXRD) and simulation of single crystal.


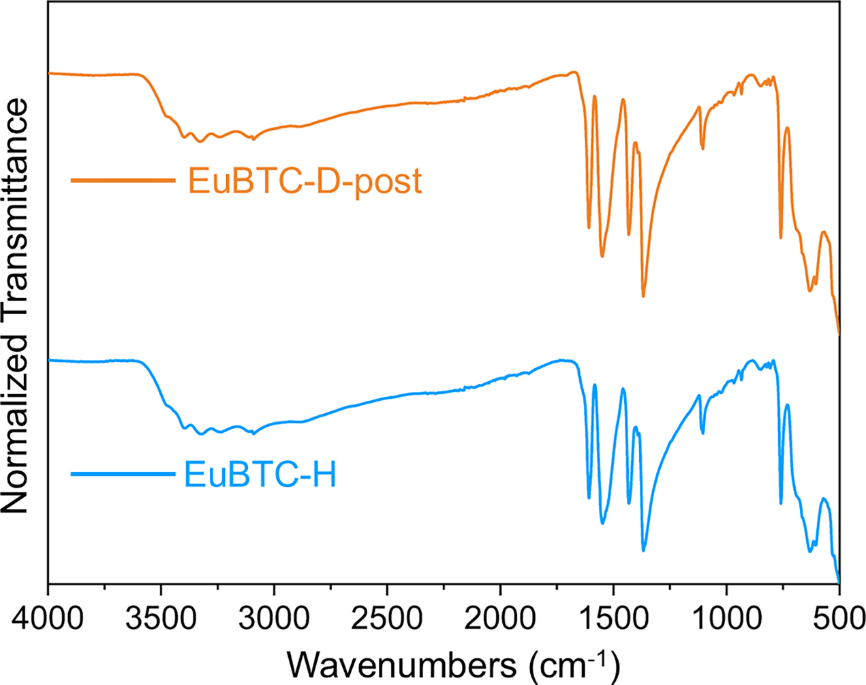


**Figure S6.** Comparison of FT-IR spectrum between EuBTC-H and EuBTC-D-post.

**
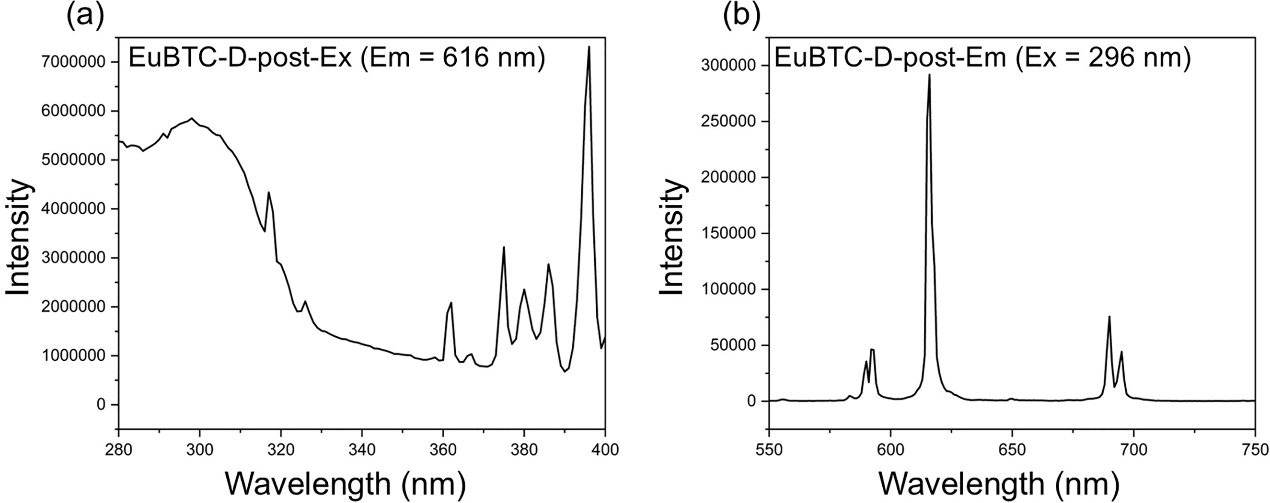
 Figure S7.** The photoluminescence (a) excitation and (b) emission spectra of EuBTC-D-post.


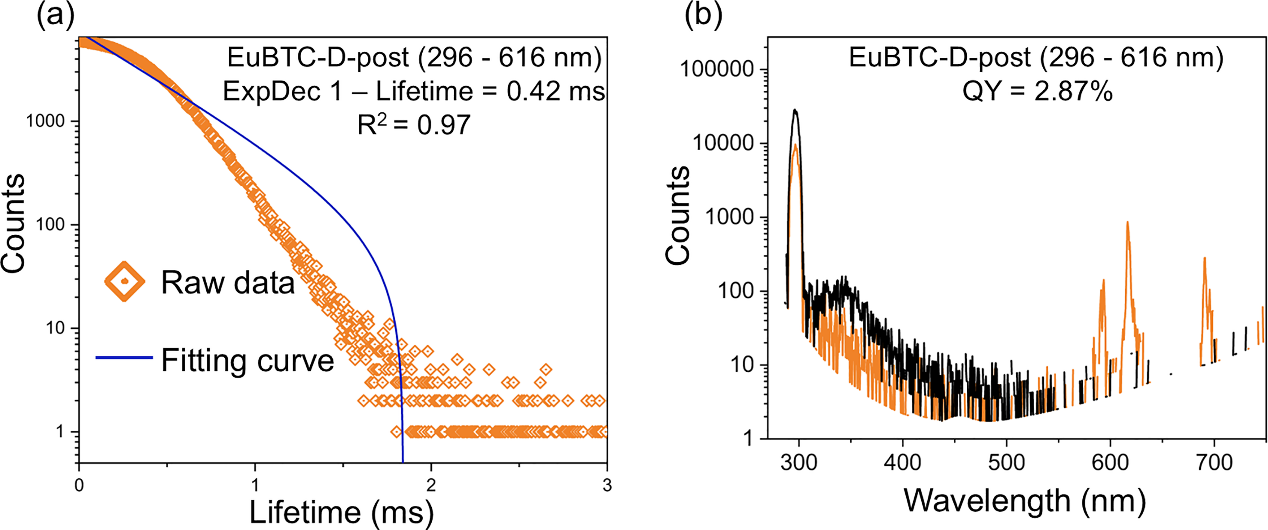


**Figure S8.** (a) Fitting of the lifetime of EuBTC-D-post based on PL data (Ex/Em = 296/616 nm, the raw data unable to fit due to the uneven exchange of water molecules). (b) Quantum yield of EuBTC-D-post based on PL data (Ex/Em = 296/616 nm).


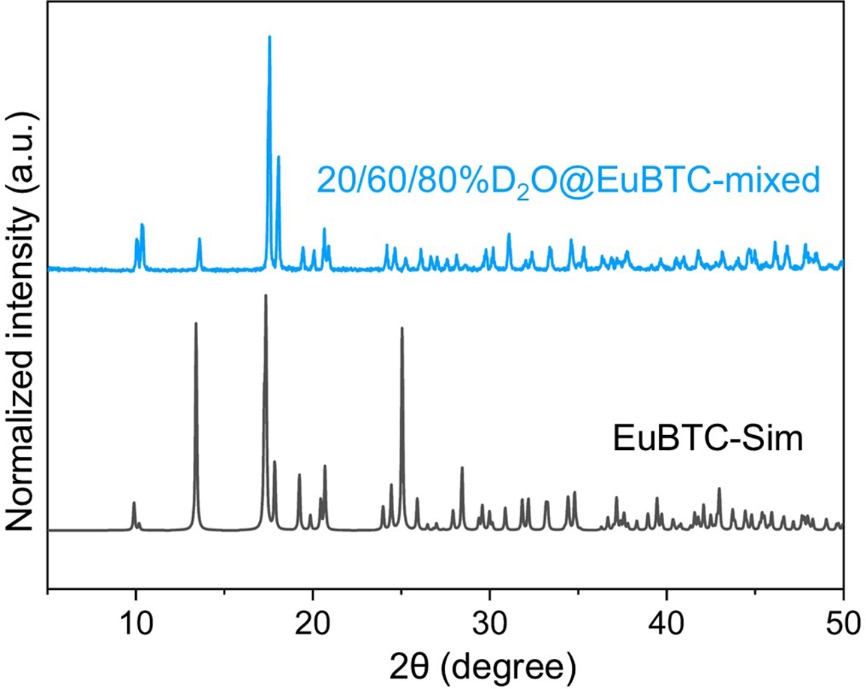


**Figure S9.** Comparison of PXRD patterns between 20/60/80%D_2_O@EuBTC-mixed and simulation of single crystal.


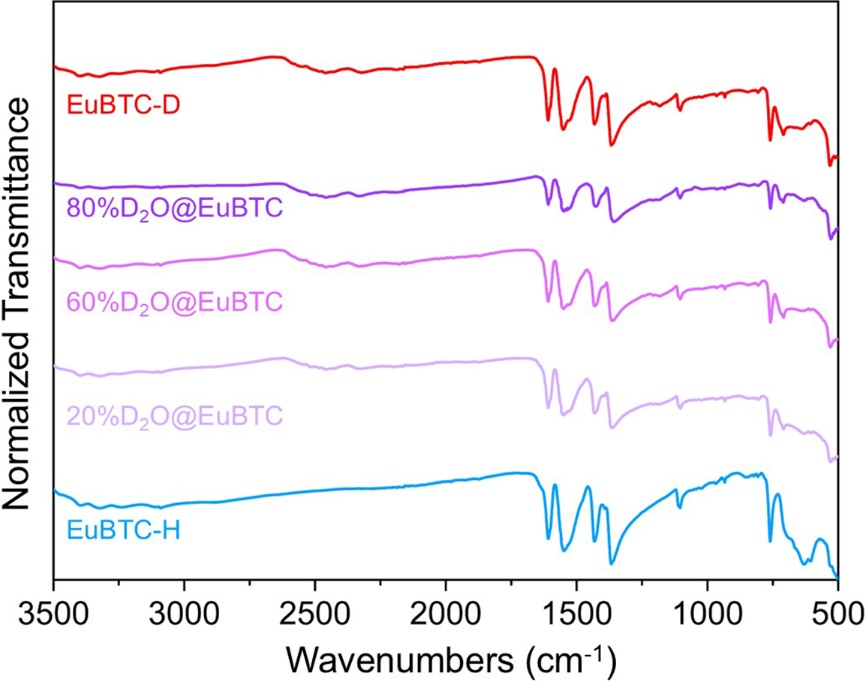


**Figure S10.** Comparison of FT-IR spectrum between 0/20/60/80/100%D_2_O@EuBTC.


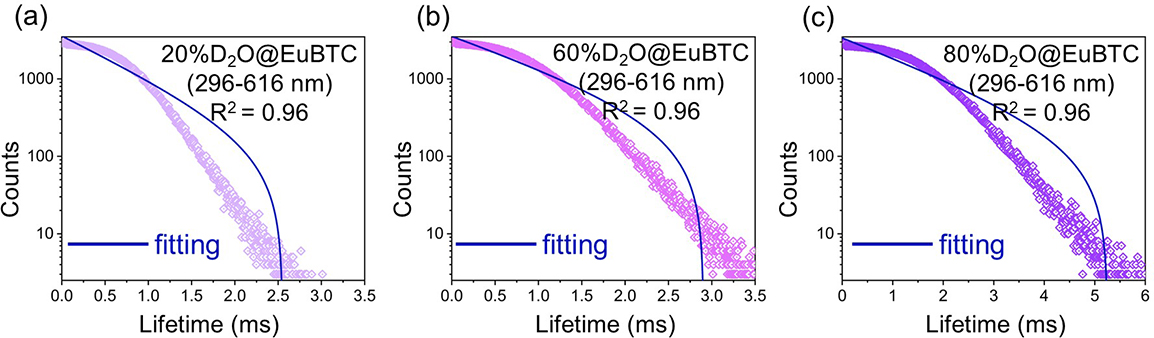


**Figure S11.** (a) Fitting of the lifetime of 20%D_2_O@EuBTC based on PL data (Ex/Em = 296/616 nm). (b) Fitting of the lifetime of 60%D_2_O@EuBTC based on PL data (Ex/Em = 296/616 nm). (c) Fitting of the lifetime of 80%D_2_O@EuBTC based on PL data (Ex/Em = 296/616 nm). The raw data unable to fit due to the uneven exchange of water molecules


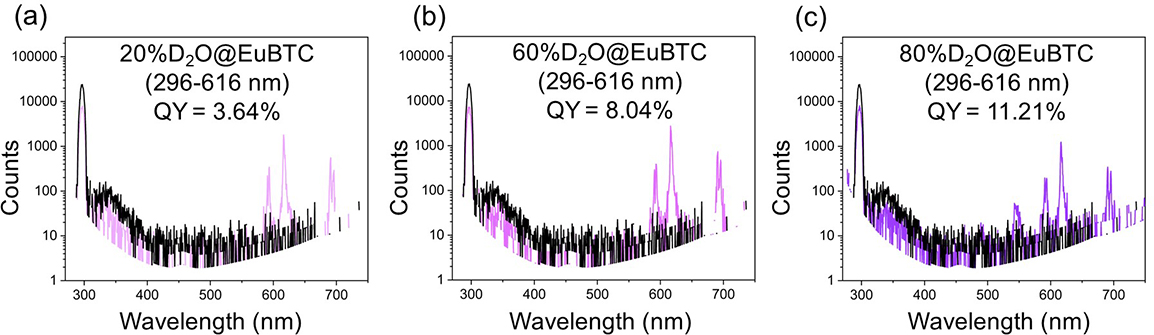


**Figure S12.** (a) Quantum yield of 20%D_2_O@EuBTC based on PL data (Ex/Em = 296/616 nm, black line for background). (b) Quantum yield of 60%D_2_O@EuBTC based on PL data (Ex/Em = 296/616 nm, black line for background). (c) Quantum yield of 80%D_2_O@EuBTC based on PL data (Ex/Em = 296/616 nm, black line for background).


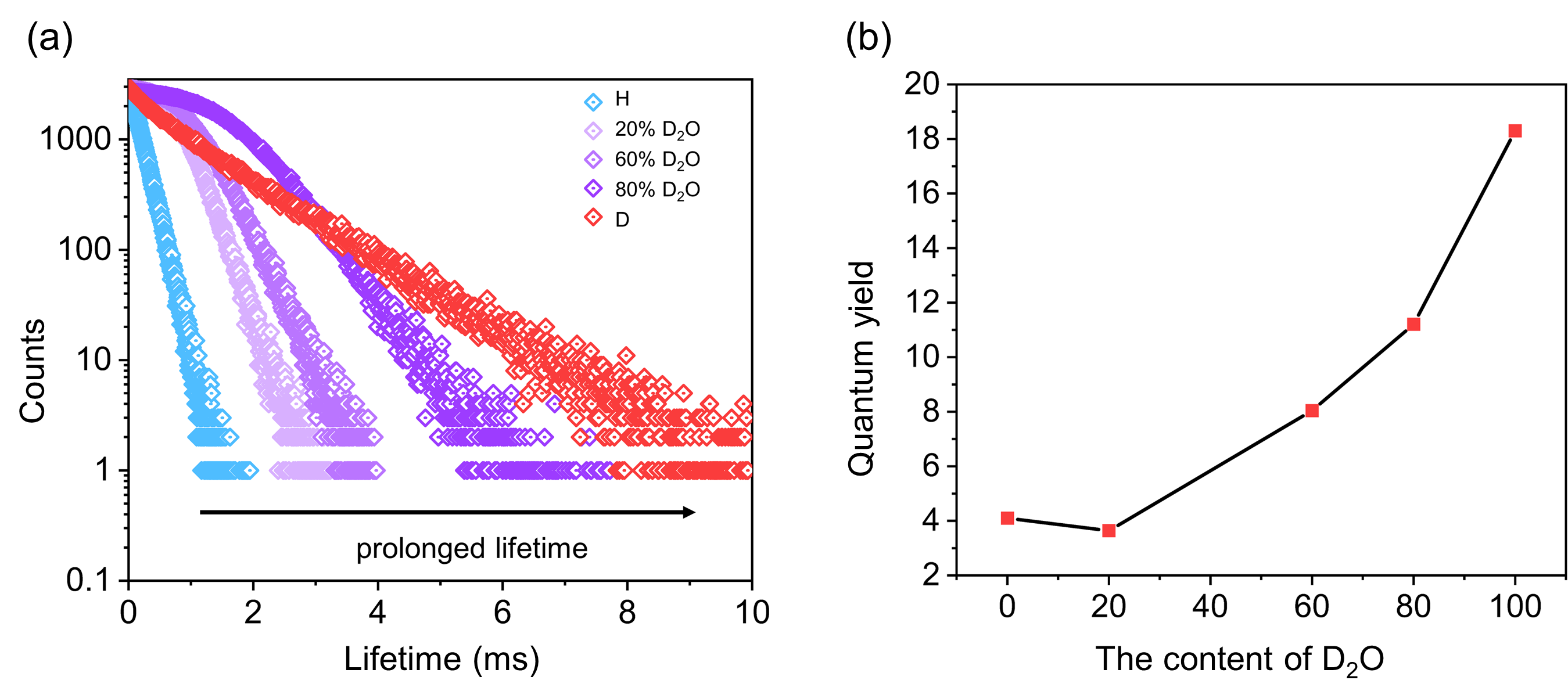


**Figure S13.** (g) The lifetime comparison of 0/20/60/80/100%D_2_O@EuBTC based on PL data (Ex/Em = 296/616 nm). (h) Comparison of quantum yield between 0/20/60/80/100%D_2_O@EuBTC in terms of the content of D_2_O.


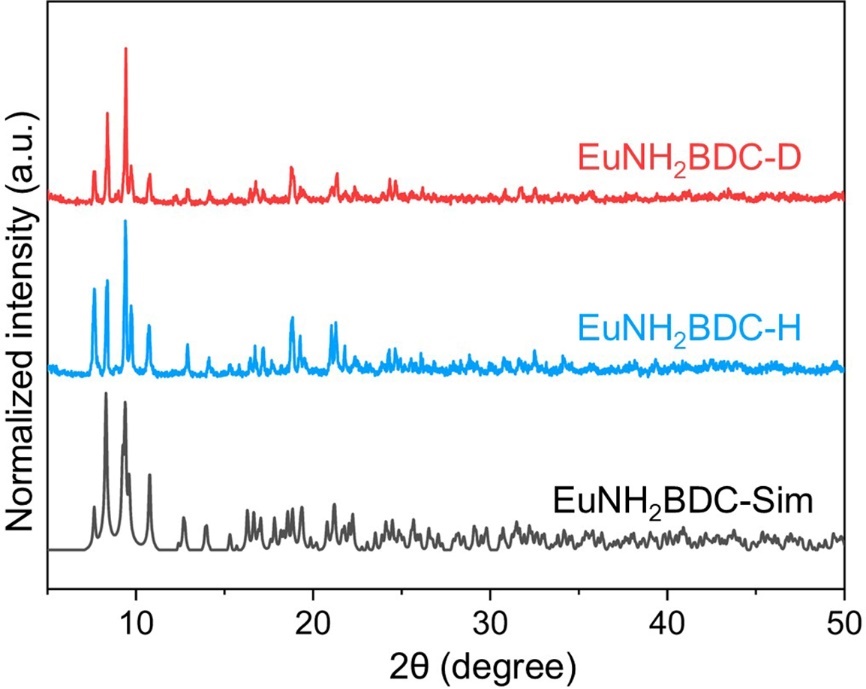


**Figure S14.** Comparison of PXRD patterns between EuNH_2_BDC-H/D and simulation of single crystal.


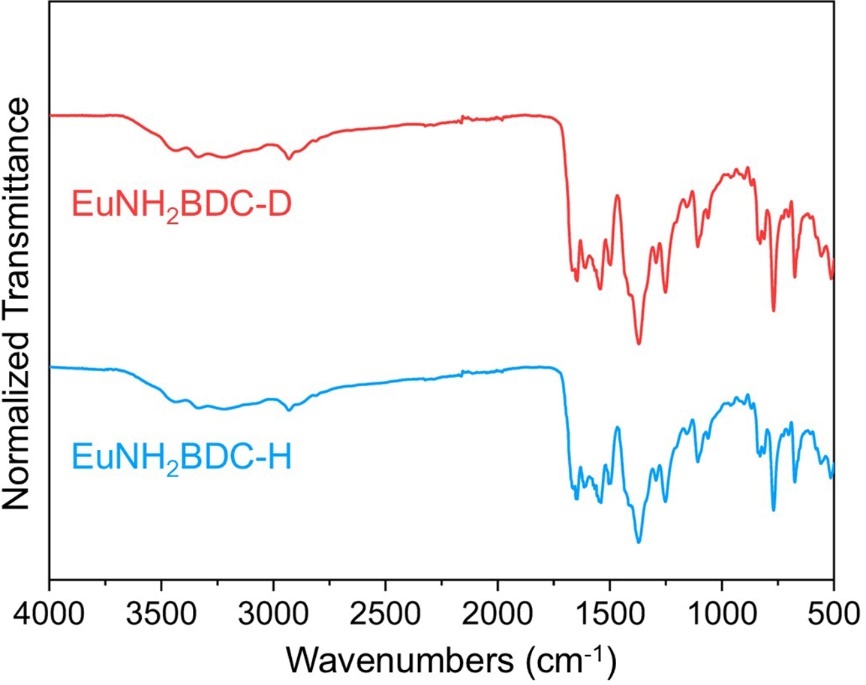


**Figure S15.** Comparison of FT-IR spectrum between EuNH_2_BDC-H/D.


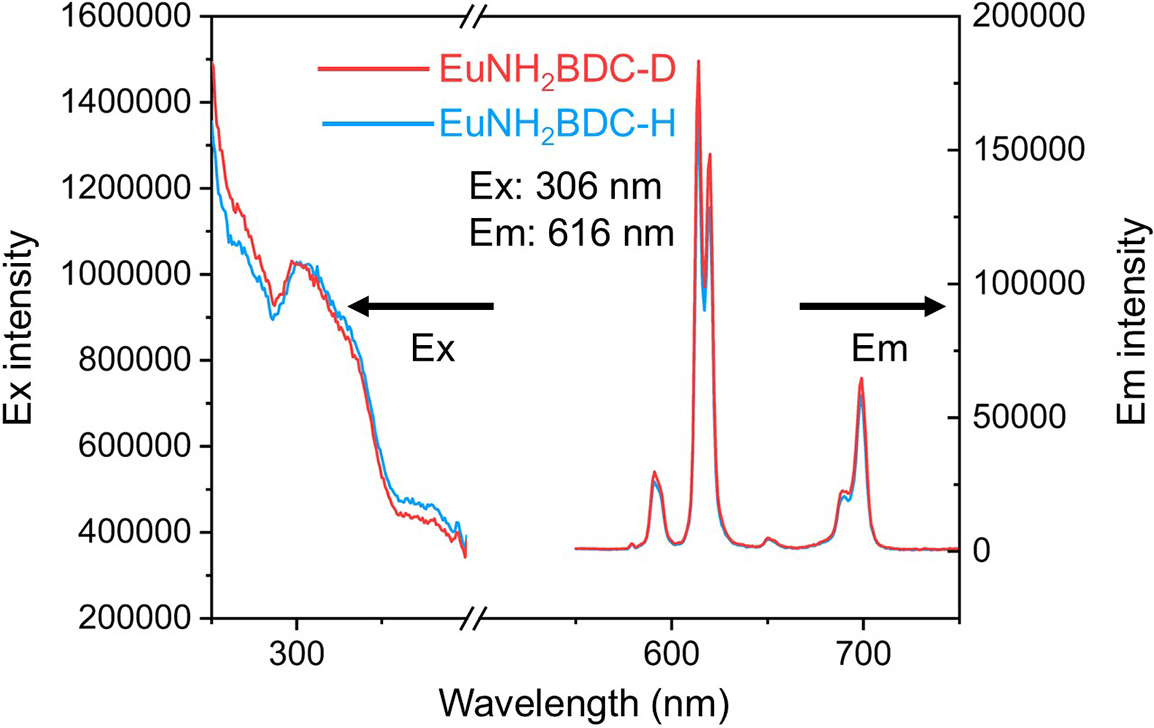


**Figure S16.** The photoluminescence excitation and emission spectra of EuNH_2_BDC-H/D.


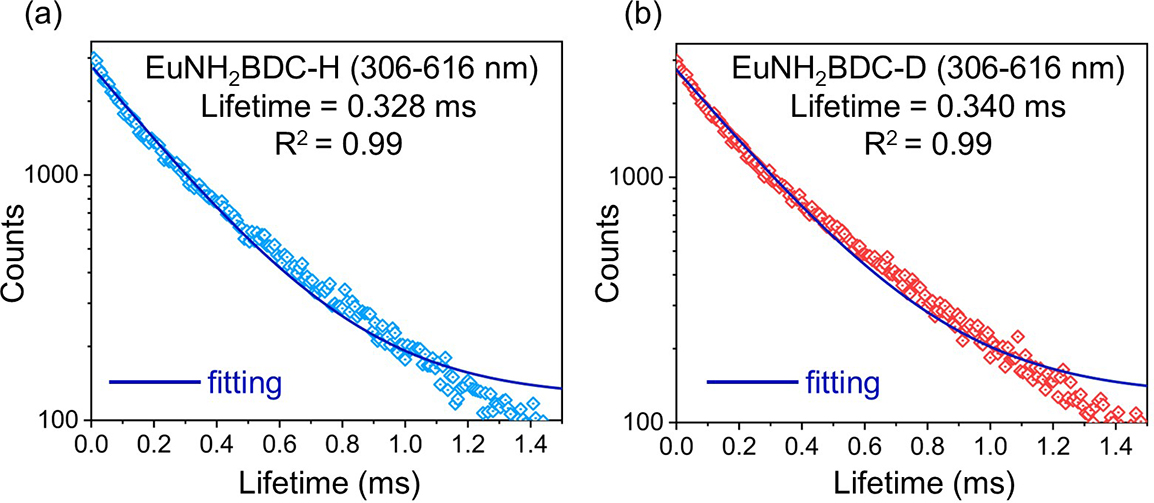


**Figure S17.** Fitting of the lifetime of EuNH_2_BDC-H/D based on PL data (Ex/Em = 306/616 nm).


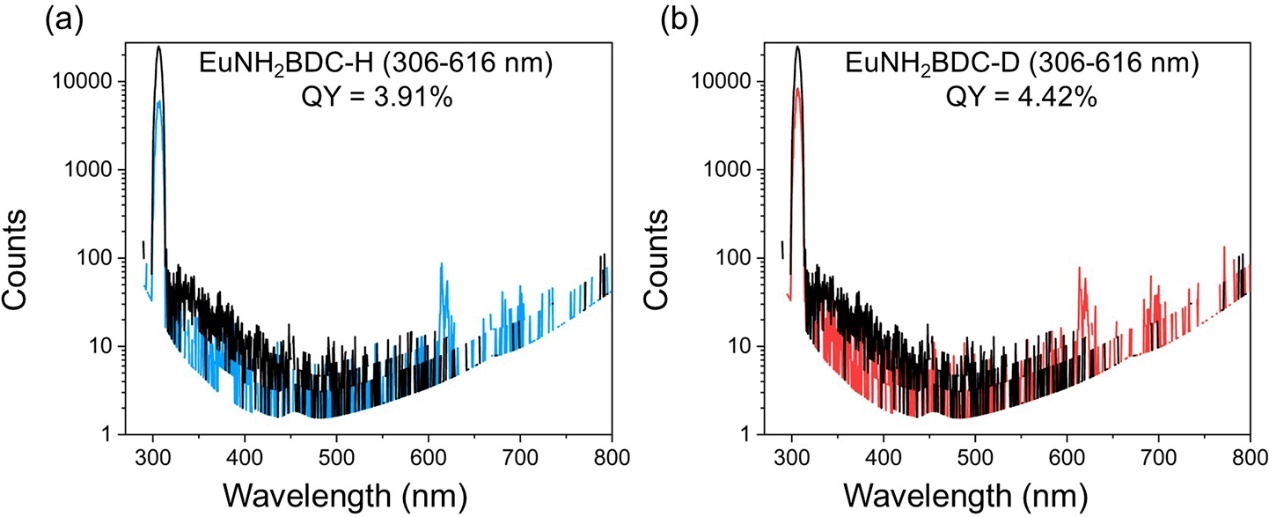


**Figure S18.** Quantum yield of EuNH_2_BDC-H/D based on PL data (Ex/Em = 306/616 nm, blue line for H, red line for D, black line for background).


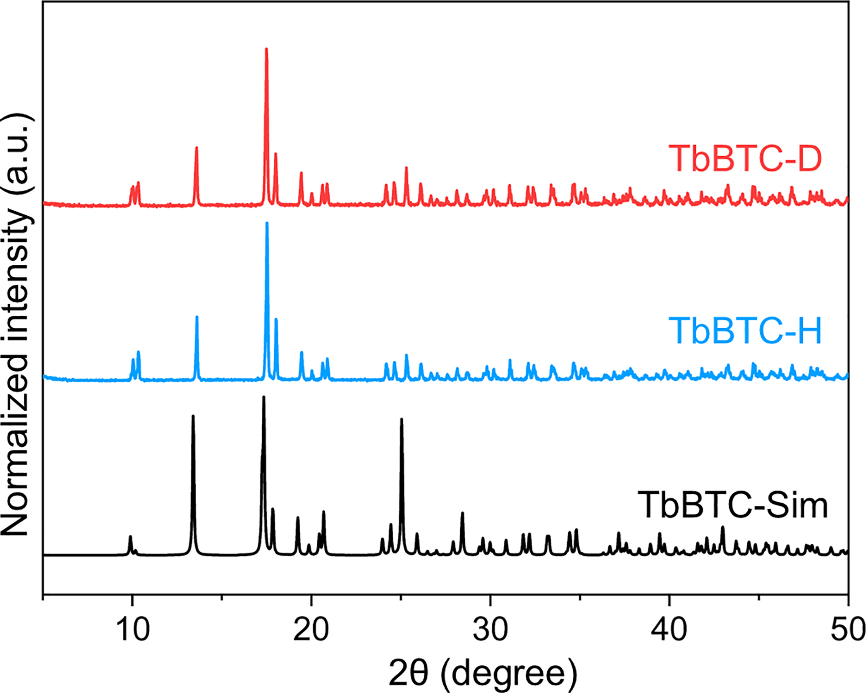


**Figure S19.** Comparison of PXRD patterns between TbBTC-H/D and simulation of single crystal.


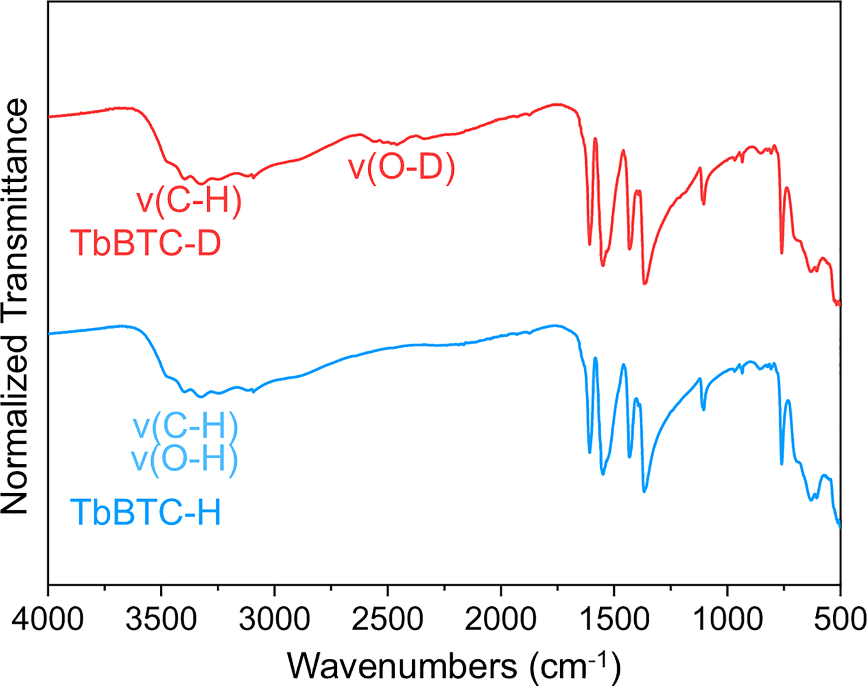


**Figure S20.** Comparison of FT-IR spectrum between TbBTC-H and TbBTC-D.


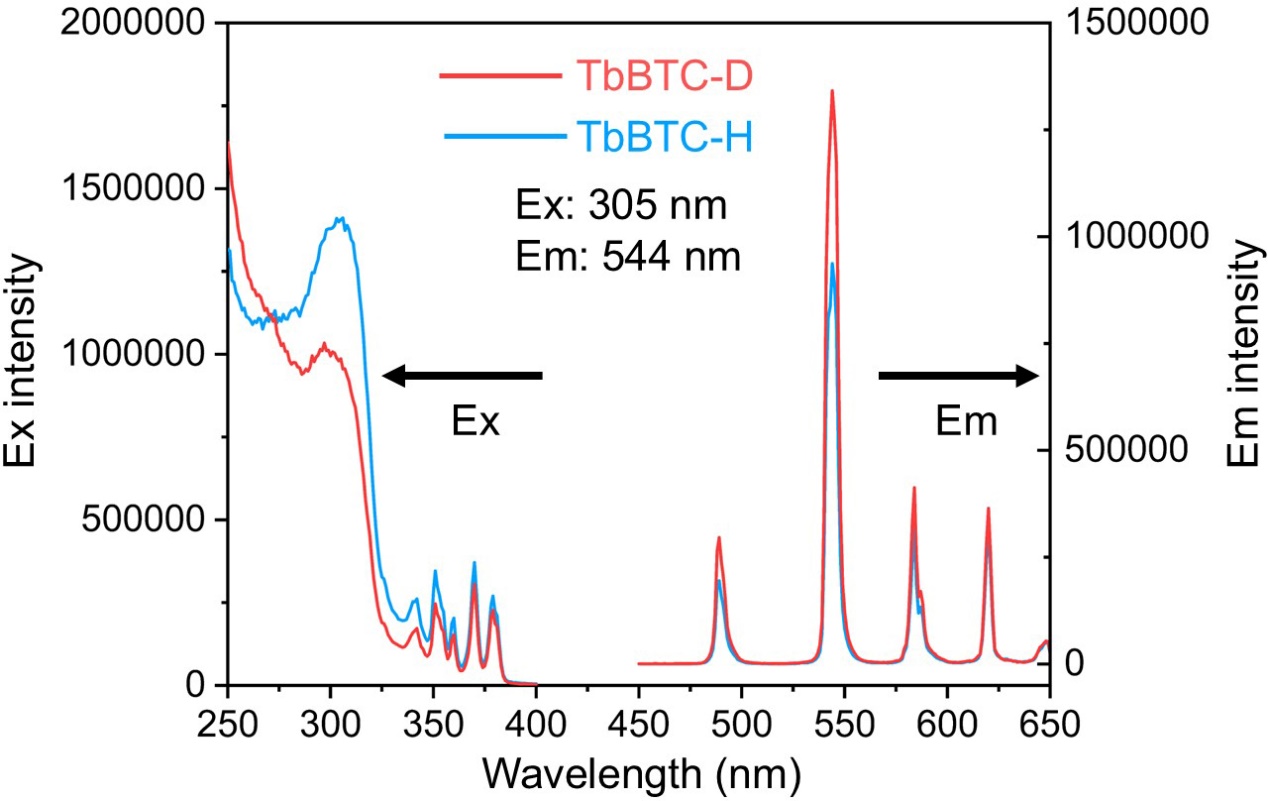


**Figure S21.** The photoluminescence excitation and emission spectra of TbBTC-H/D.


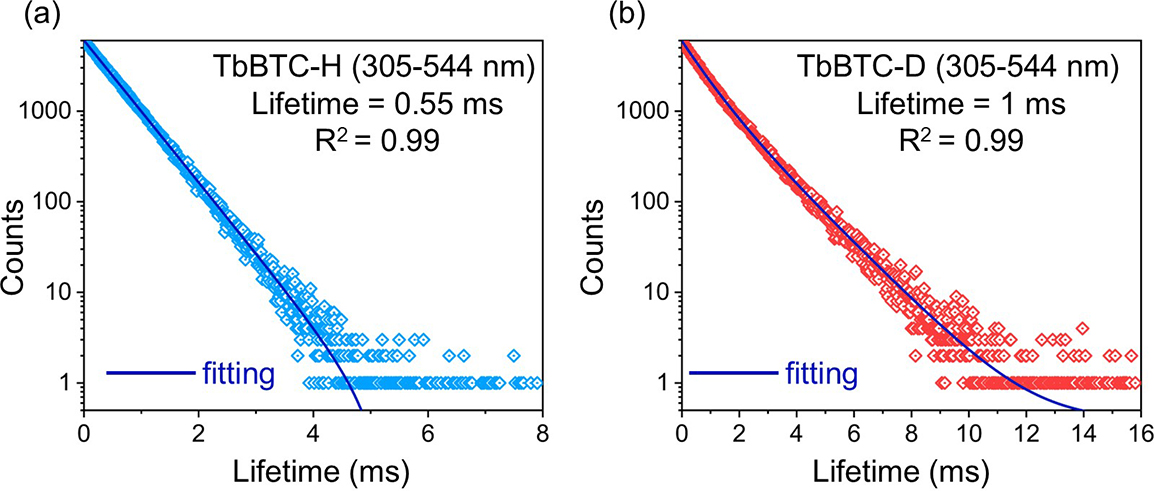


**Figure S22.** Fitting of the lifetime of TbBTC-H/D based on PL data (Ex/Em = 305/544 nm).


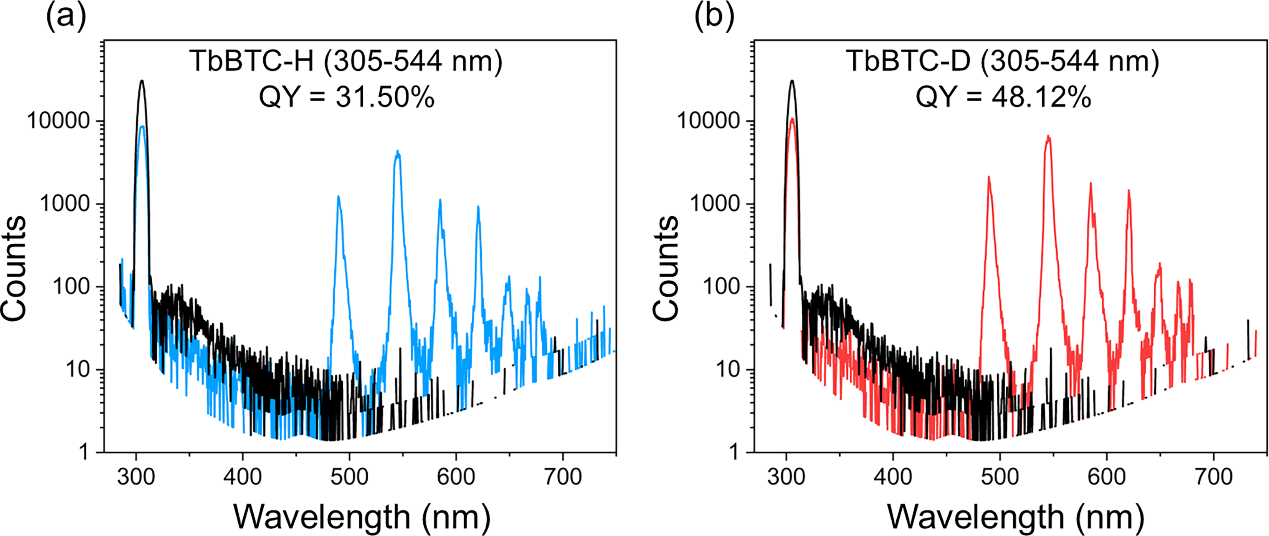


**Figure S23.** Quantum yield of TbBTC-H/D based on PL data (Ex/Em = 305/544 nm, blue line for H, red line for D, black line for background).


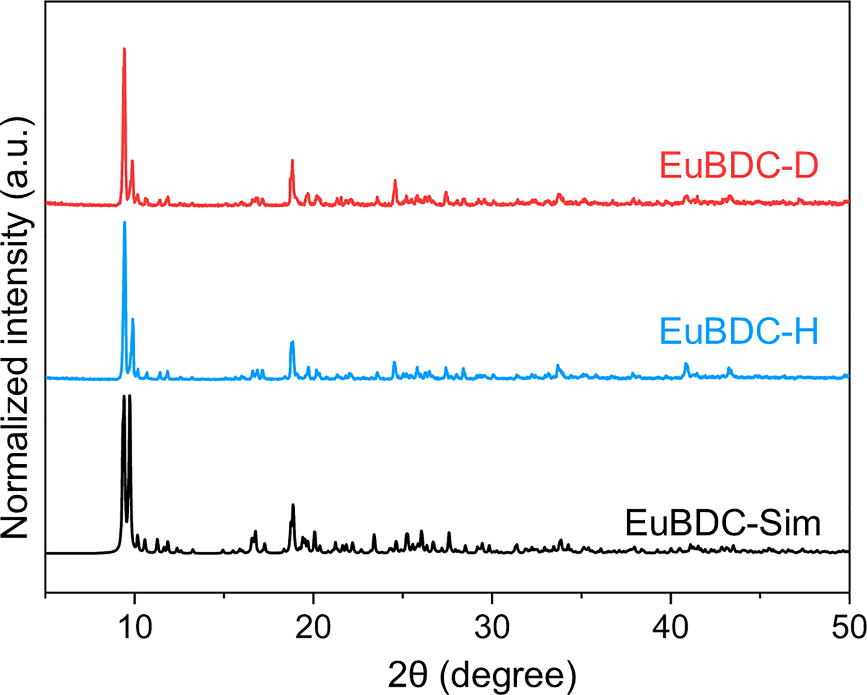


**Figure S24.** Comparison of PXRD patterns between EuBDC-H/D and simulation of single crystal.


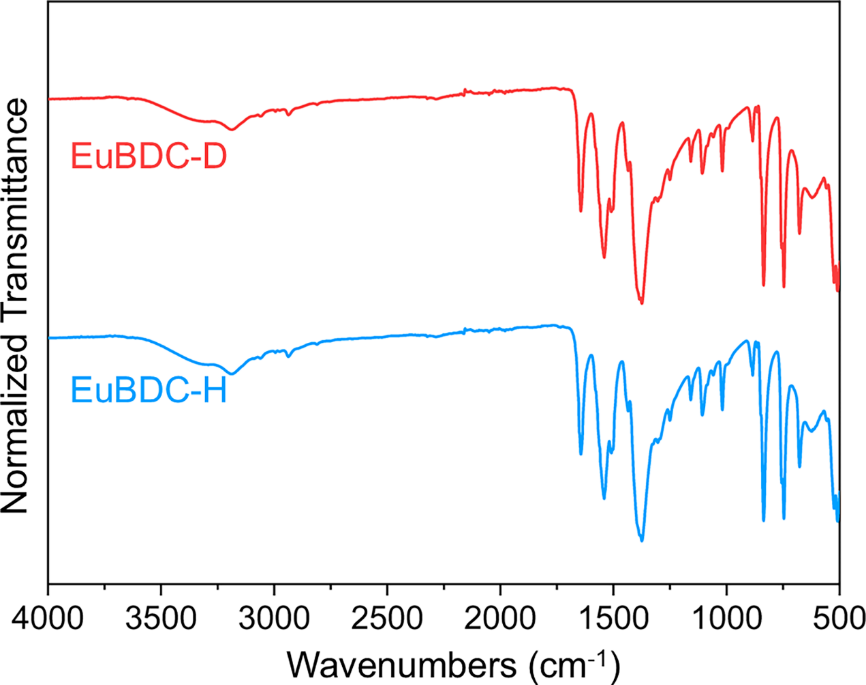


**Figure S25.** Comparison of FT-IR spectrum between EuBDC-H and EuBDC-D.


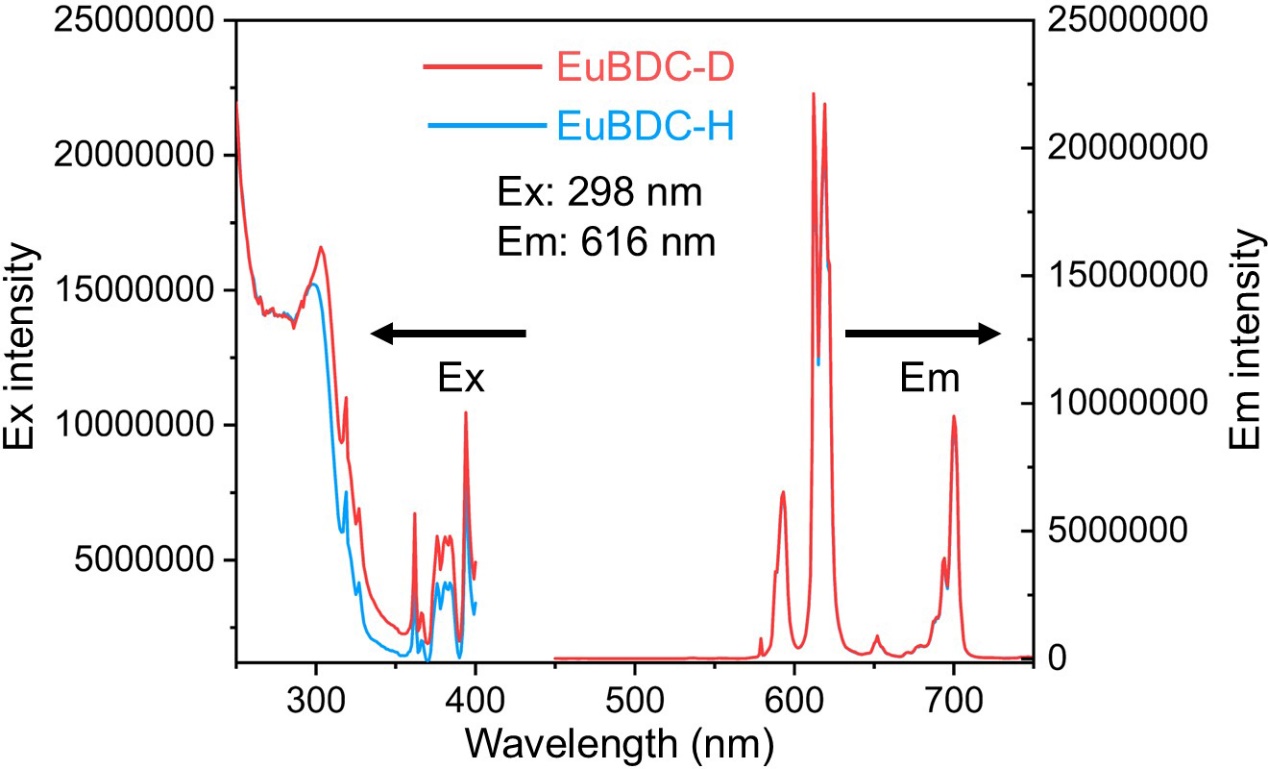


**Figure S26.** The photoluminescence excitation and emission spectra of EuBDC-H/D.


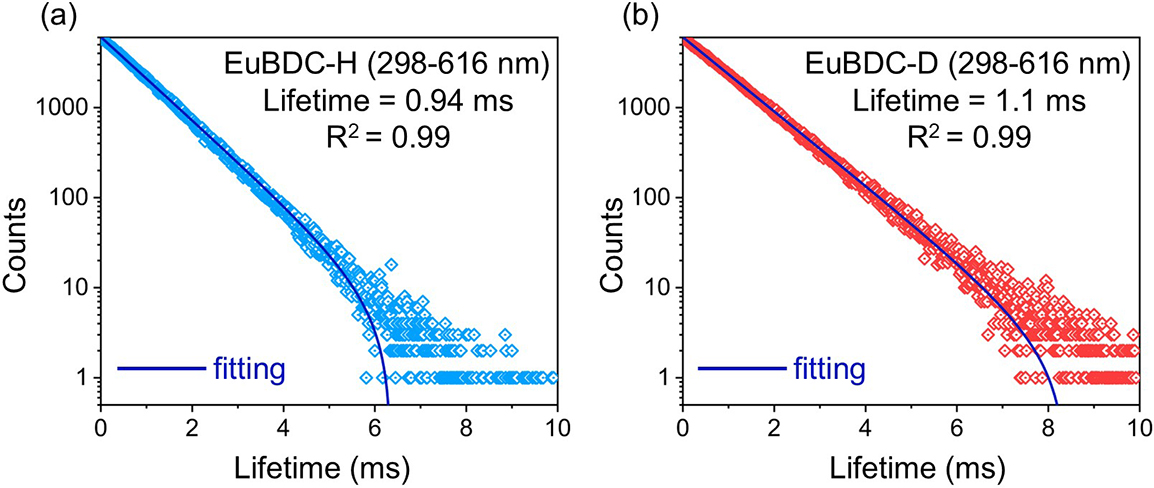


**Figure S27.** Fitting of the lifetime of EuBDC-H/D based on PL data (Ex/Em = 298/616 nm).


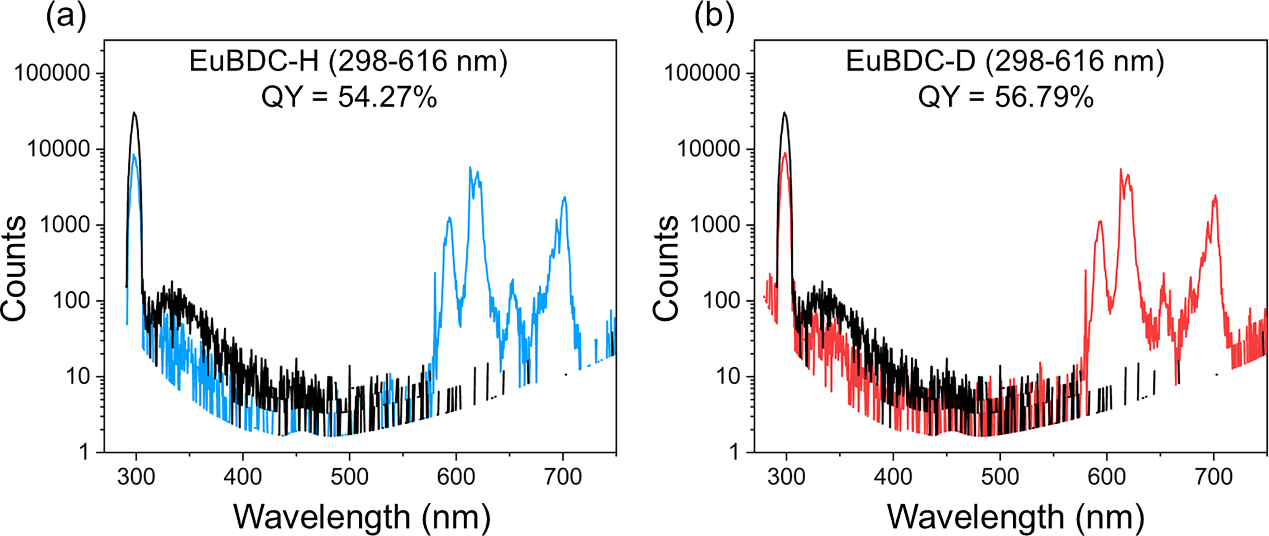


**Figure S28.** Quantum yield of EuBDC-H/D based on PL data (Ex/Em = 298/616 nm, blue line for H, red line for D, black line for background).


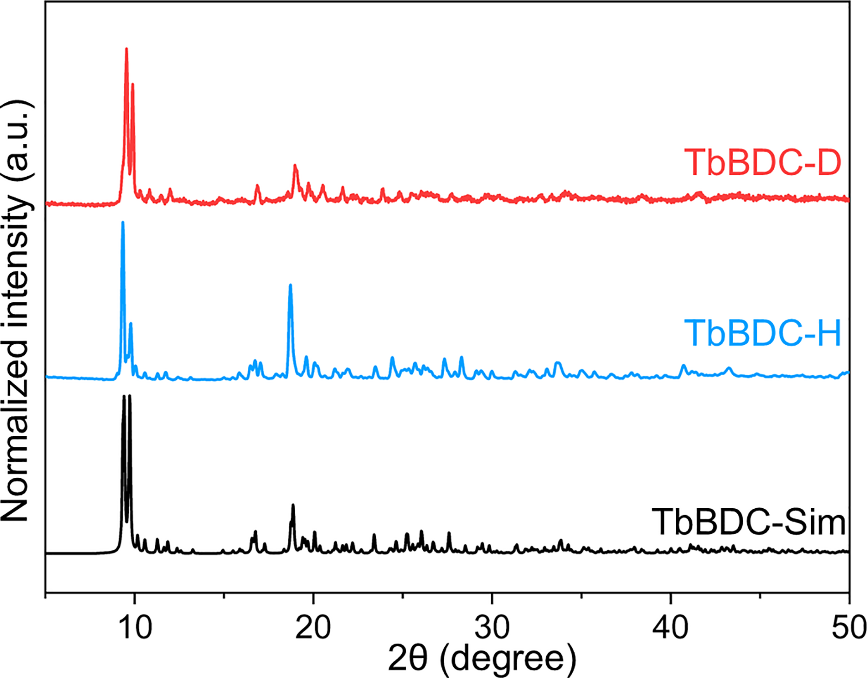


**Figure S29.** Comparison of PXRD patterns between TbBDC-H/D and simulation of single crystal.


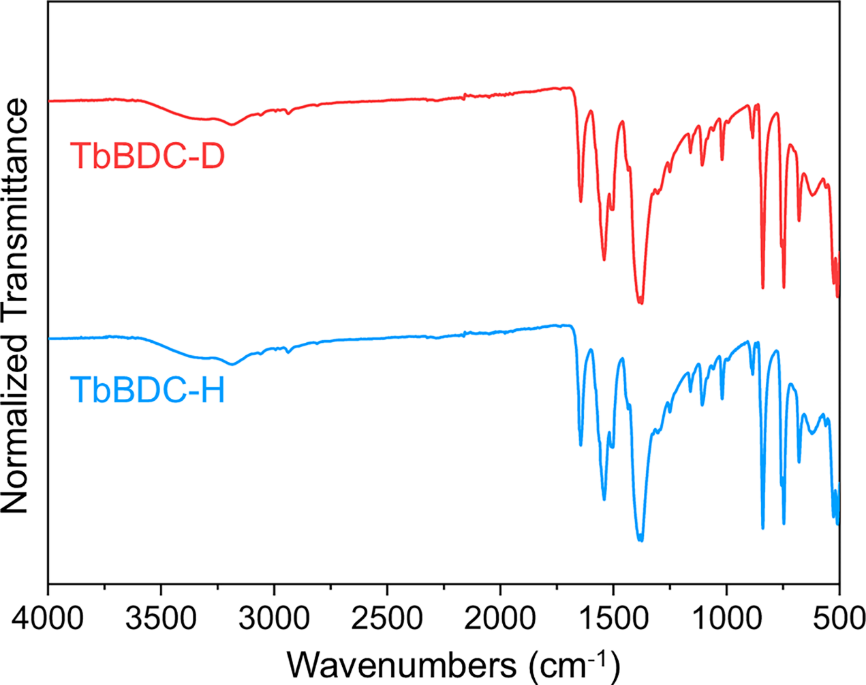


**Figure S30.** Comparison of FT-IR spectrum between TbBDC-H and TbBDC-D.


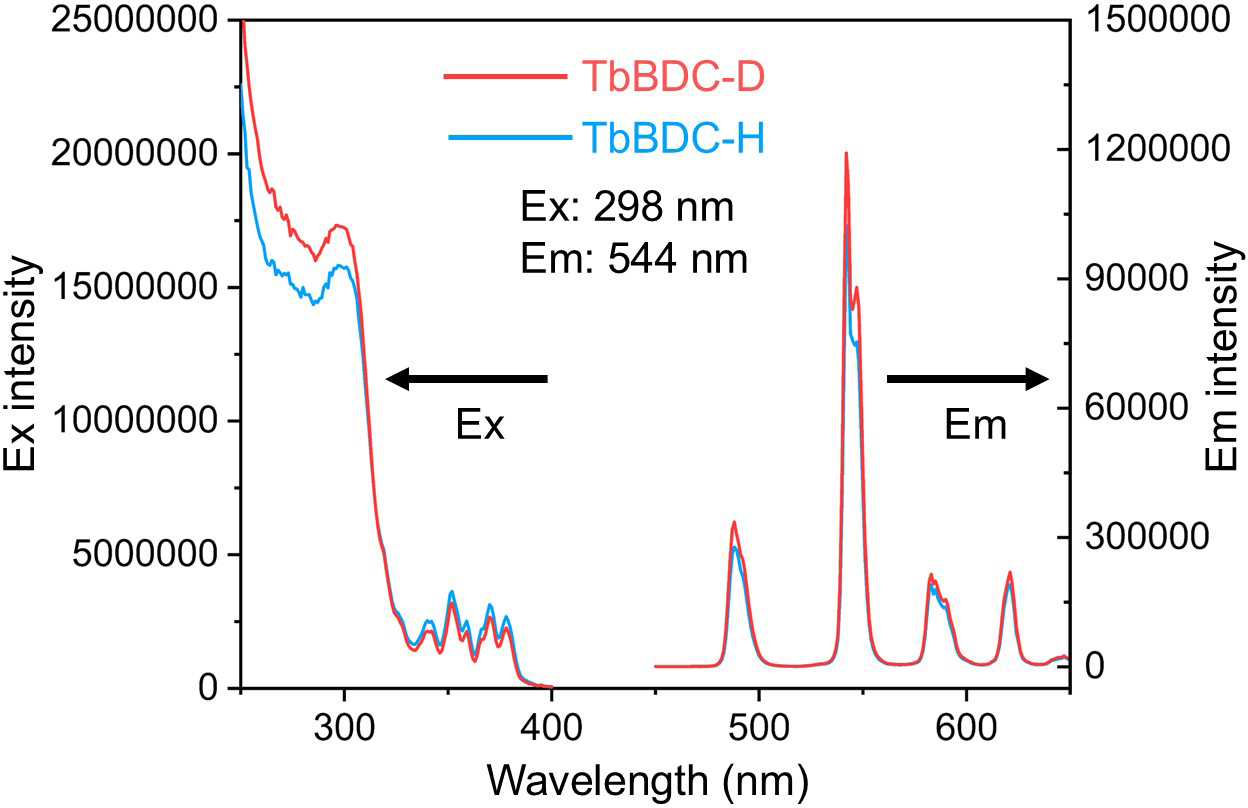


**Figure S31.** The photoluminescence excitation and emission spectra of TbBDC-H/D.


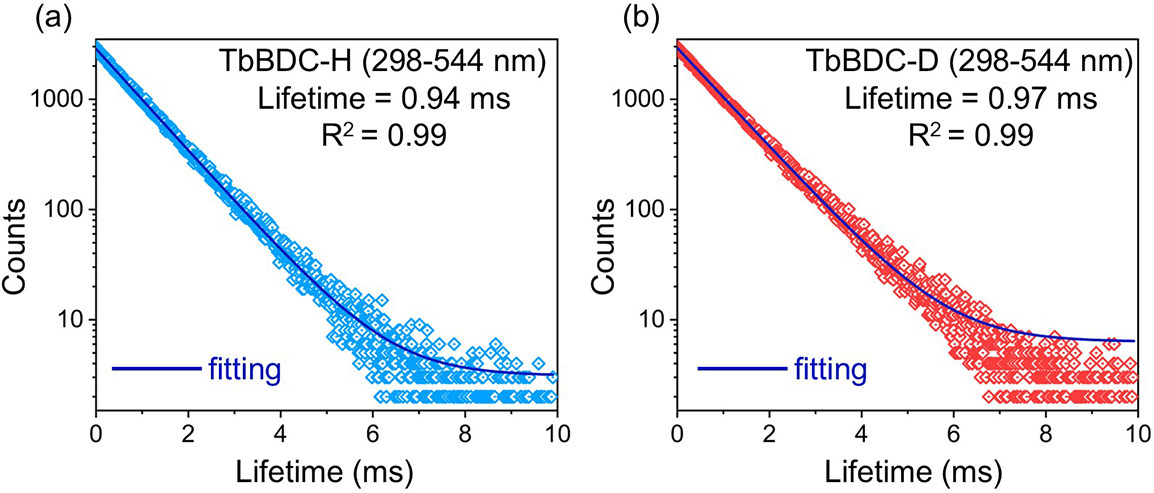


**Figure S32.** Fitting of the lifetime of TbBDC-H/D based on PL data (Ex/Em = 298/544 nm).


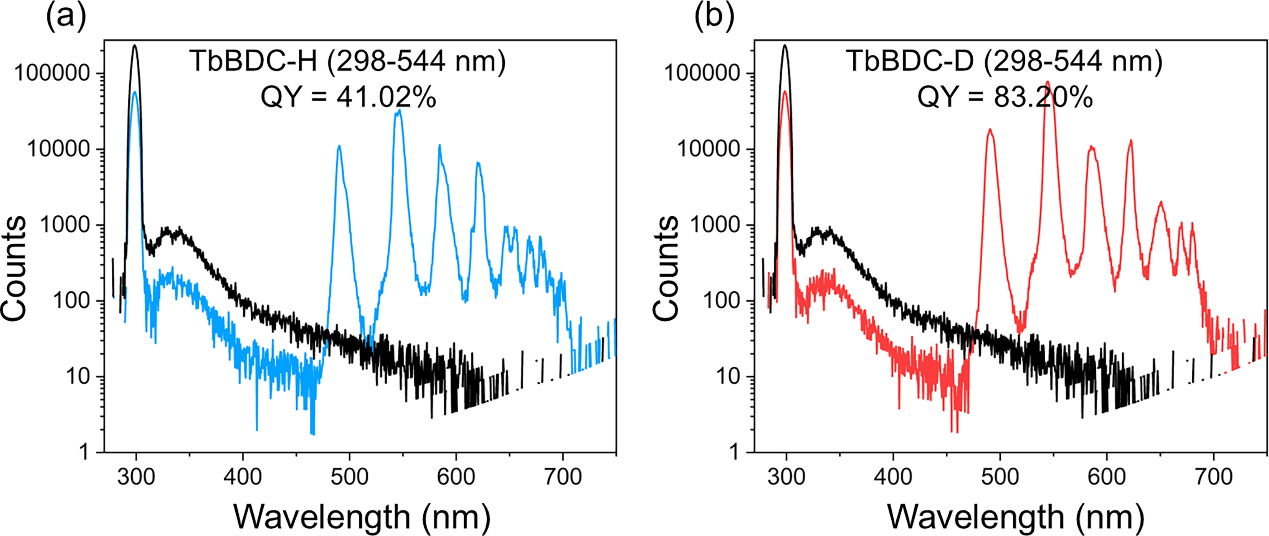


**Figure S33.** Quantum yield of TbBDC-H/D based on PL data (Ex/Em = 298/544 nm, blue line for H, red line for D, black line for background).


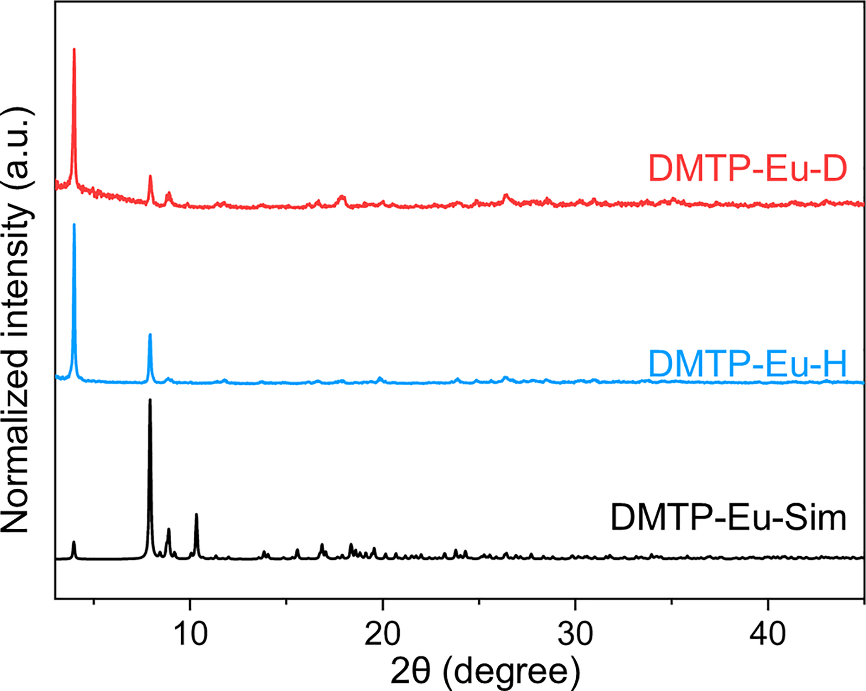


**Figure S34.** Comparison of PXRD patterns between DMTPEu-H/D and simulation of single crystal.


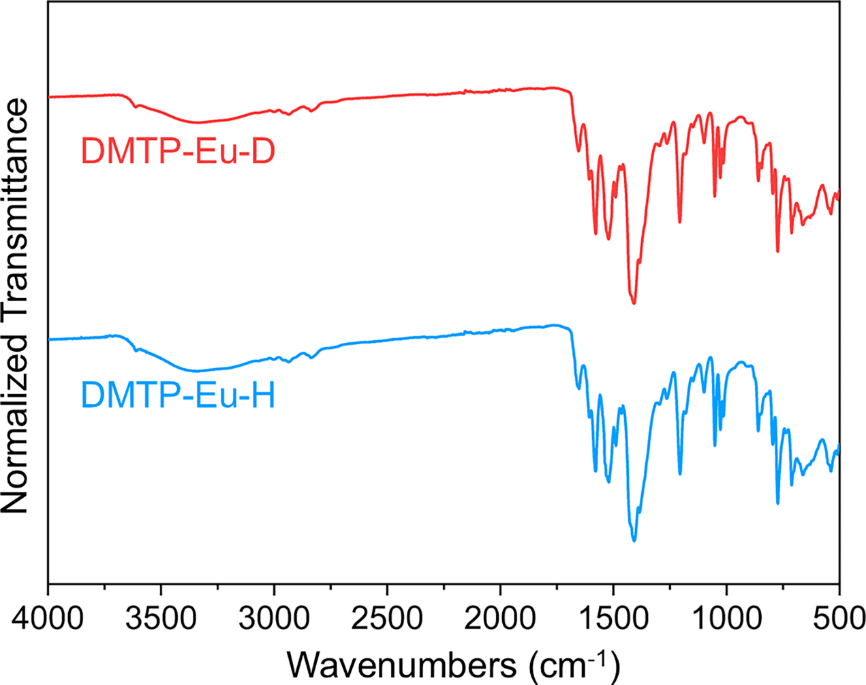


**Figure S35.** Comparison of FT-IR spectrum between DMTPEu-H and DMTPEu-D.


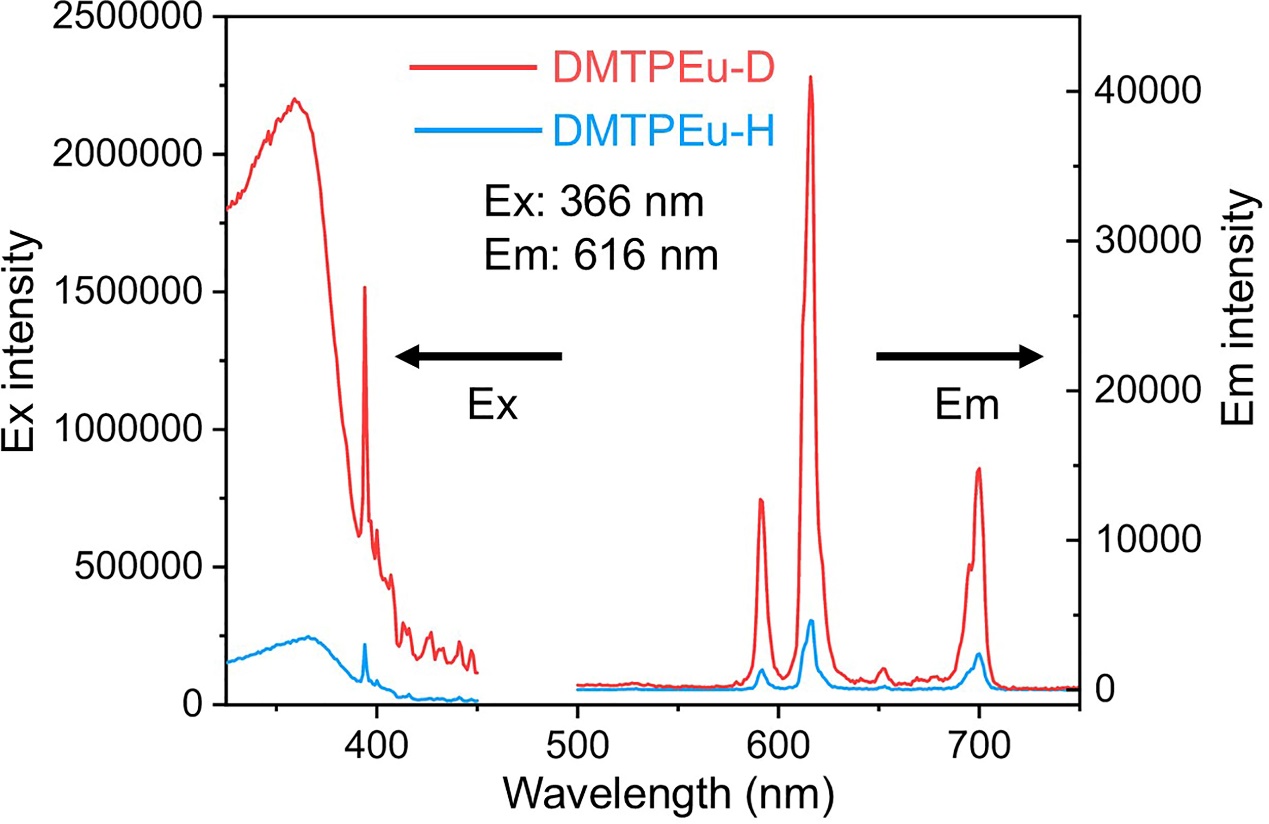


**Figure S36.** The photoluminescence excitation and emission spectra of DMTPEu-H/D.


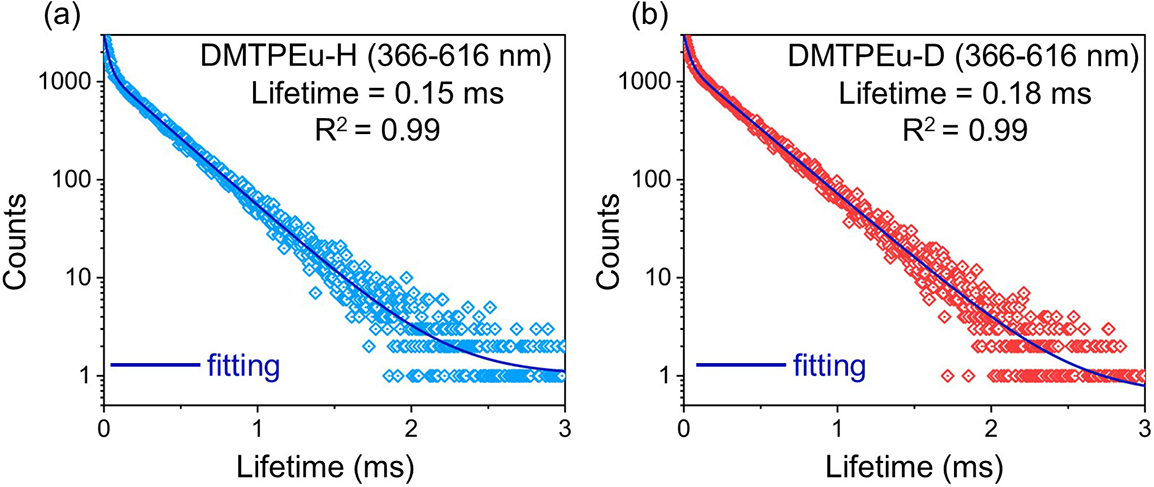


**Figure S37.** Fitting of the lifetime of DMTPEu-H/D based on PL data (Ex/Em = 366/616 nm).


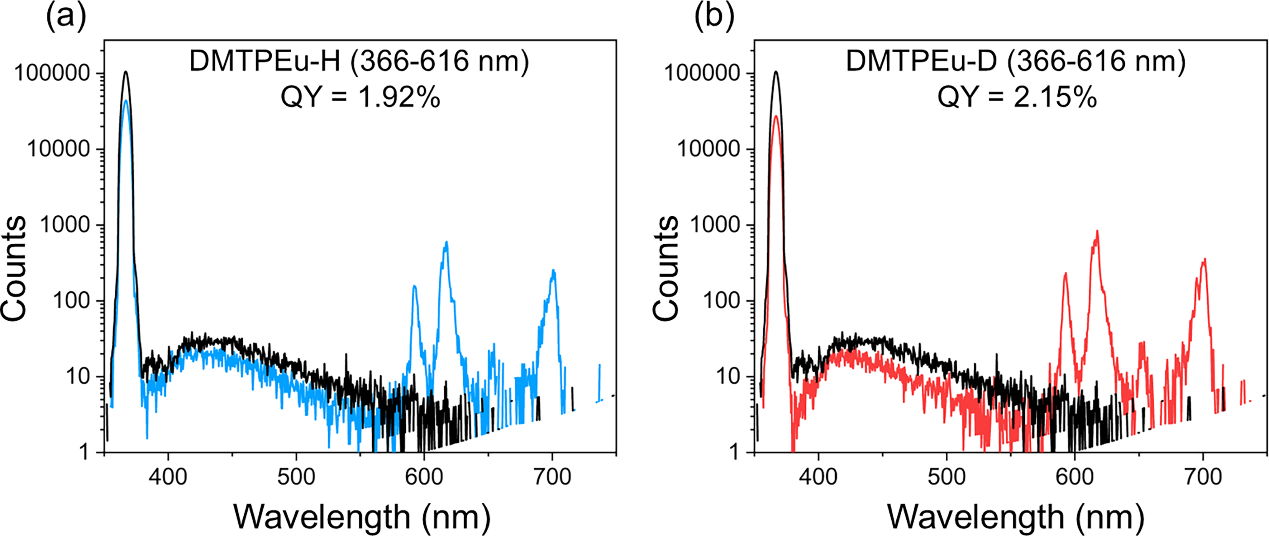


**Figure S38.** Quantum yield of DMTPEu-H/D based on PL data (Ex/Em = 366/616 nm, blue line for H, red line for D, black line for background).


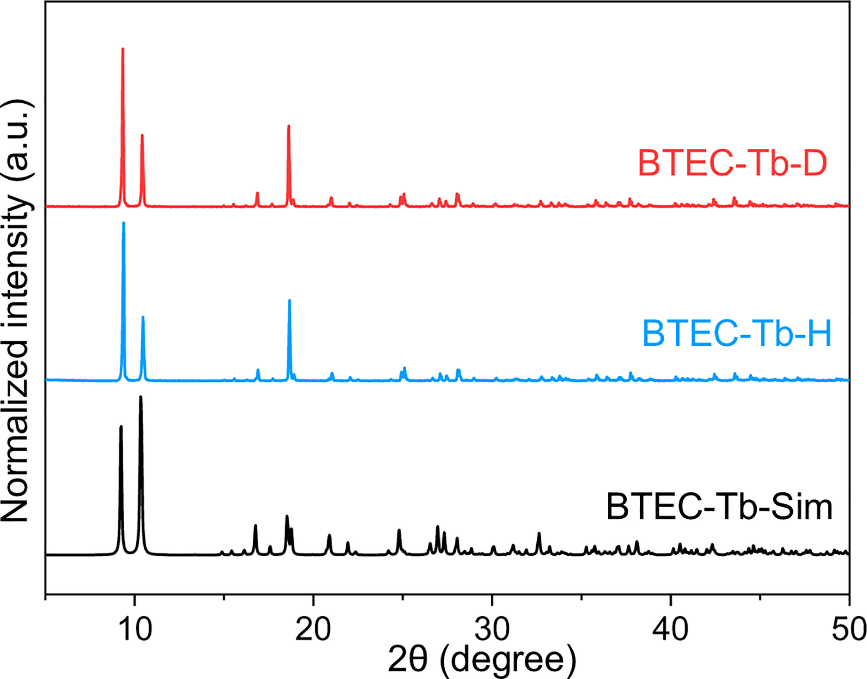


**Figure S39.** Comparison of PXRD patterns between BTECTb-H/D and simulation of single crystal.


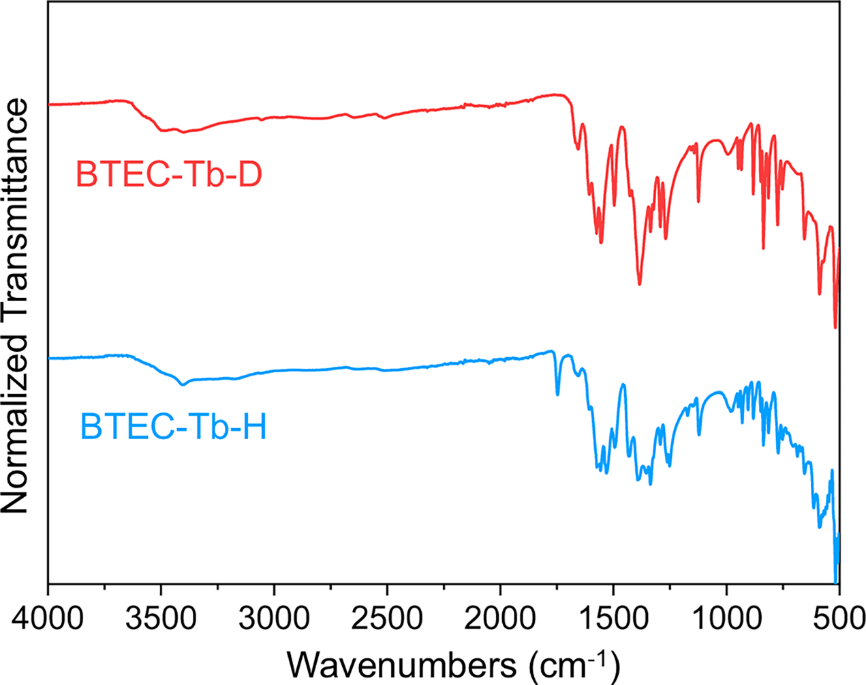


**Figure S40.** (a) Comparison of FT-IR spectrum between BTECTb-H and BTECTb-D.


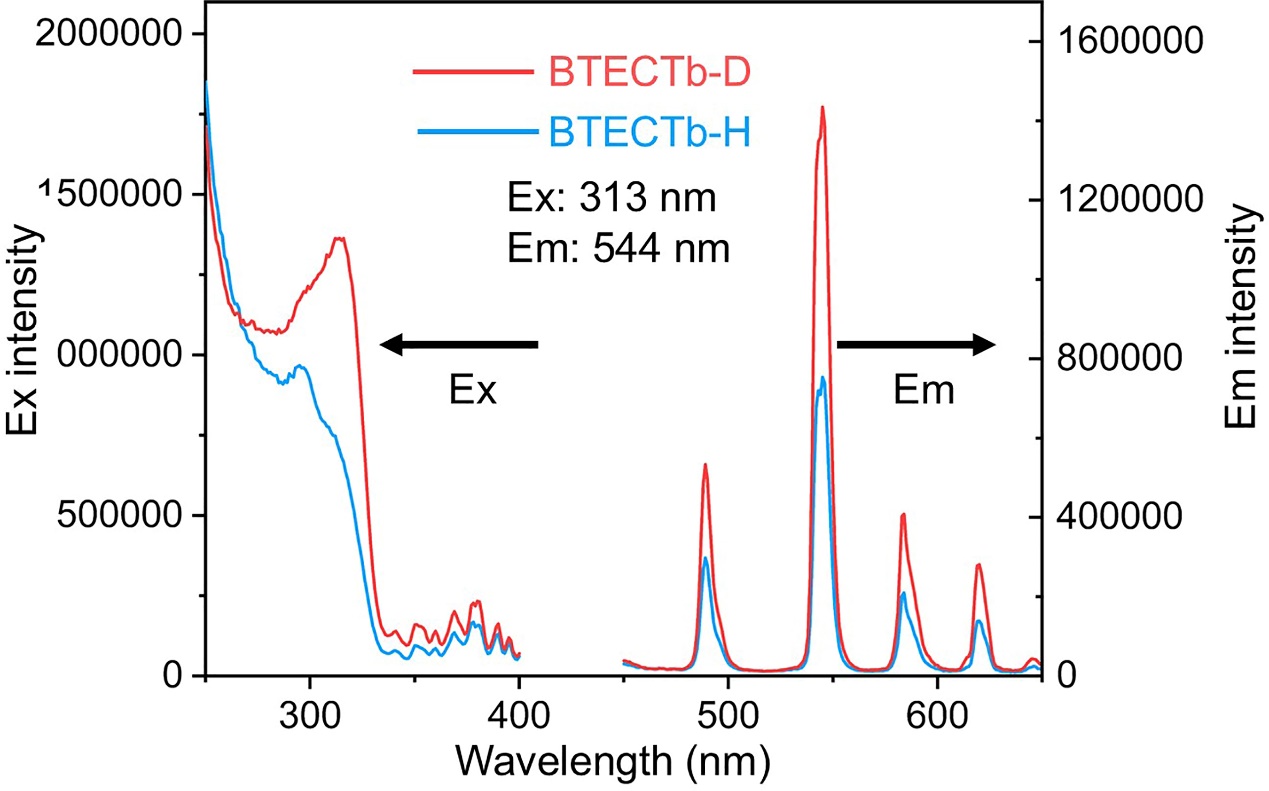


**Figure S41.** The photoluminescence excitation and emission spectra of BTECTb-H/D.


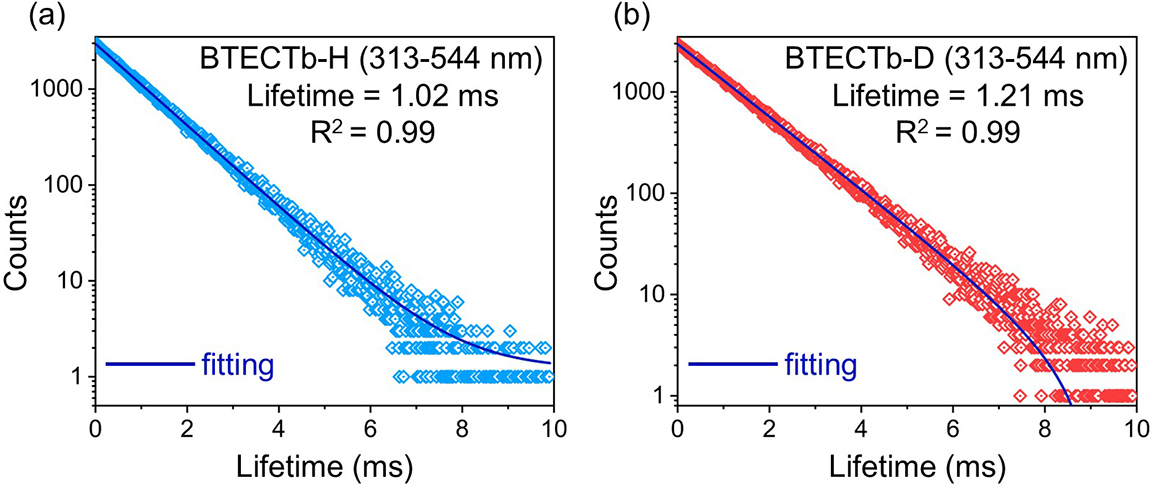


**Figure S42.** Fitting of the lifetime of BTECTb-H/D based on PL data (Ex/Em = 313/544 nm).


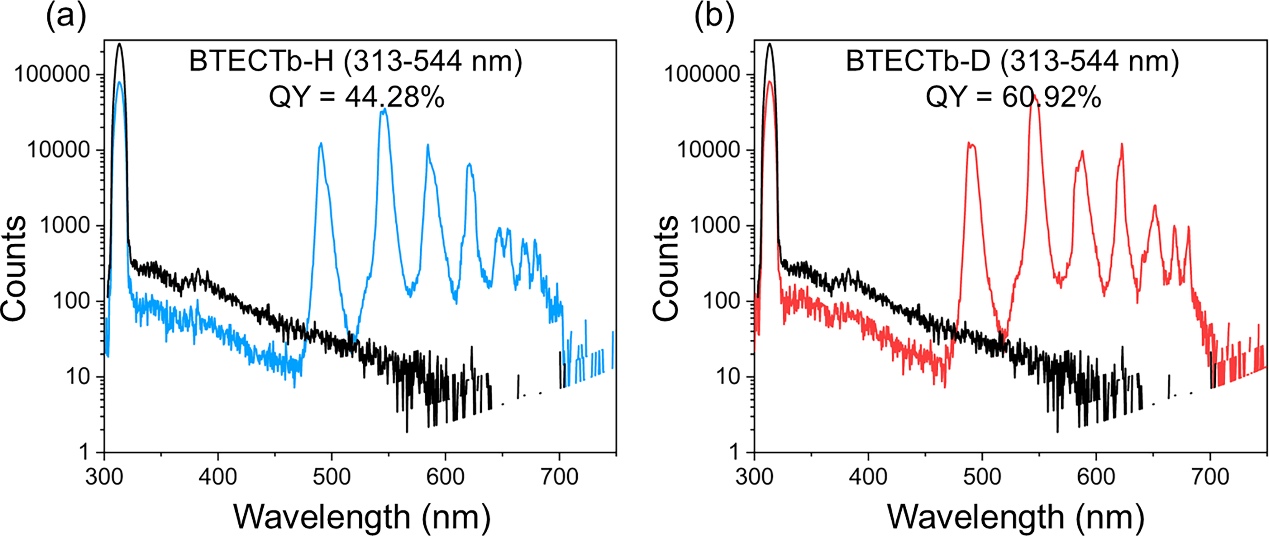


**Figure S43.** Quantum yield of BTECTb-H/D based on PL data (Ex/Em = 313/544 nm, blue line for H, red line for D, black line for background).

**
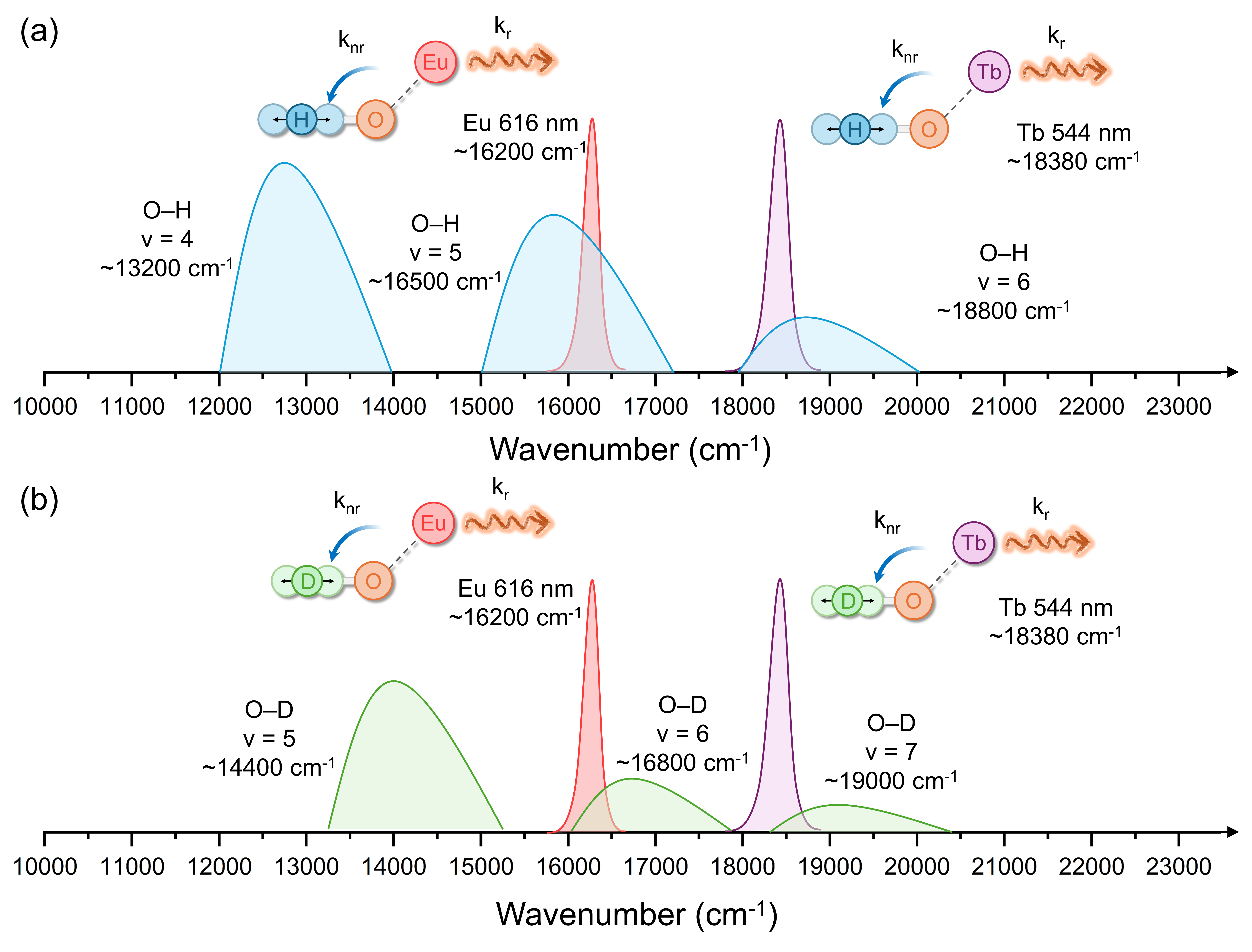
 Figure S44.** Emission spectrum of Eu^3+^ (red line) and the relevent vibrational O-H (blue line, v=4, 5, 6) and O-D (green line, v=5, 6, 7) overtones absorption bands, and the spectral overlap between them.


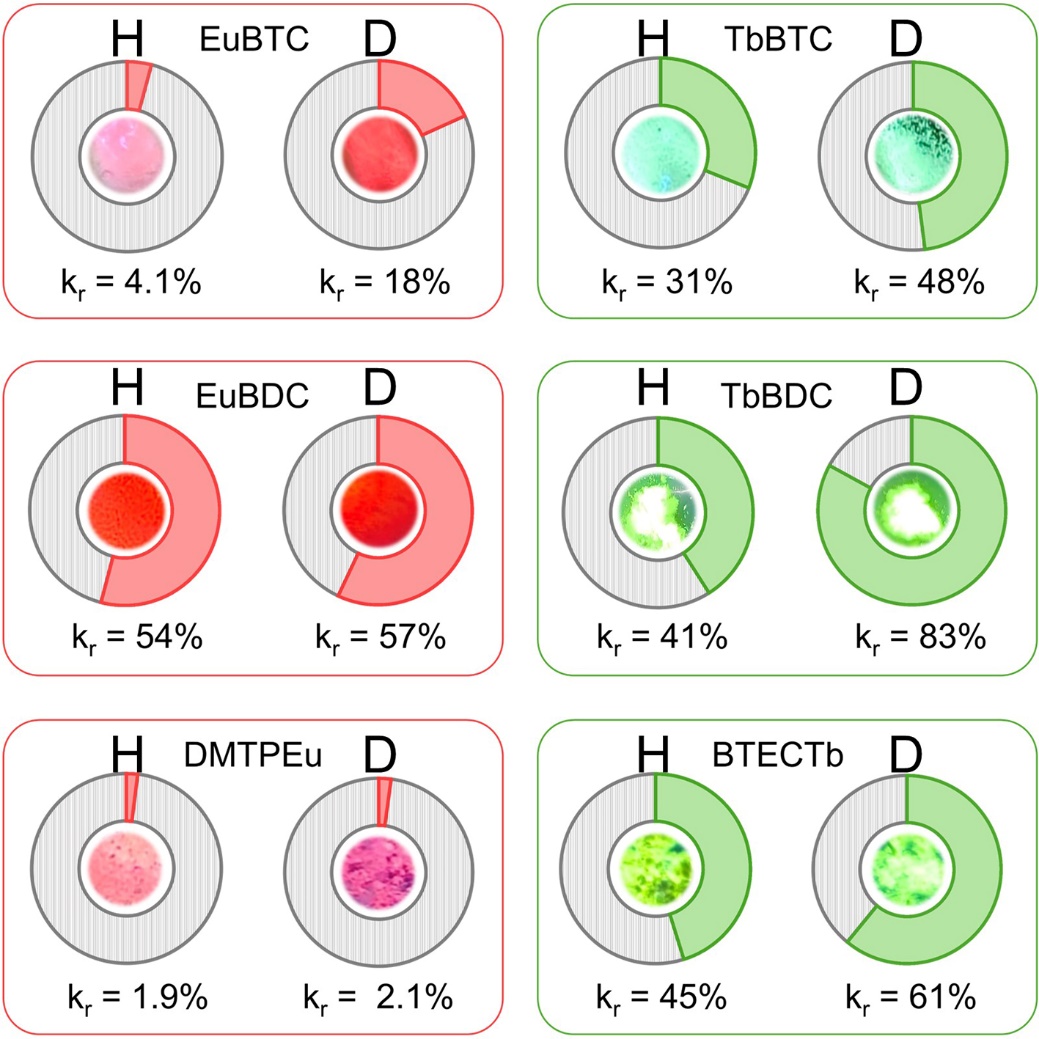


**Figure S45.** The proportion of kᵣ in k_total_ in deuterated MOFs, insets are the luminscent images under UV-irradiation.


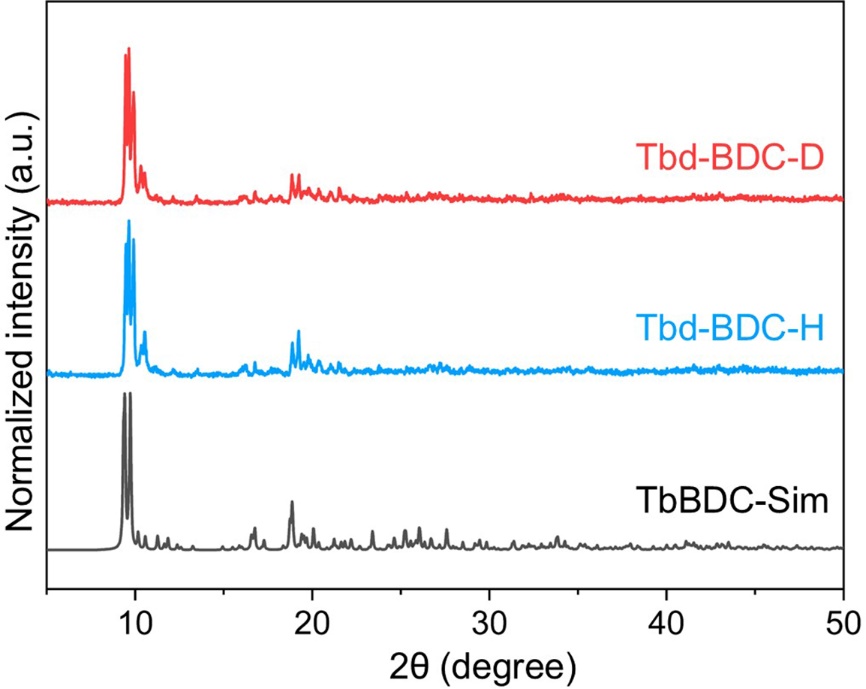


**Figure S46.** Comparison of PXRD patterns between Tbd-BDC-H/D and simulation of single crystal.


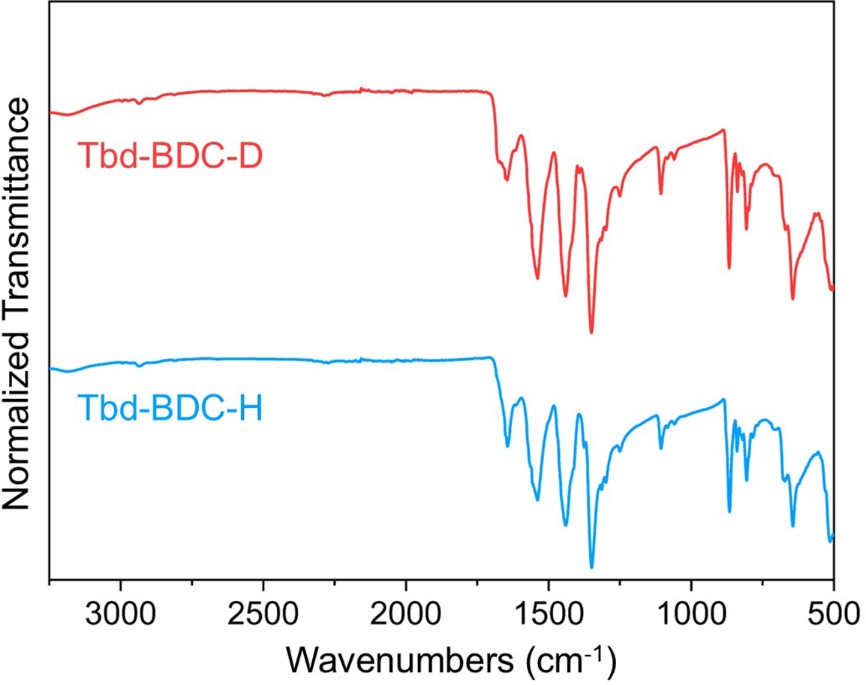


**Figure S47.** Comparison of FT-IR spectrum between Tbd-BDC-H/D.


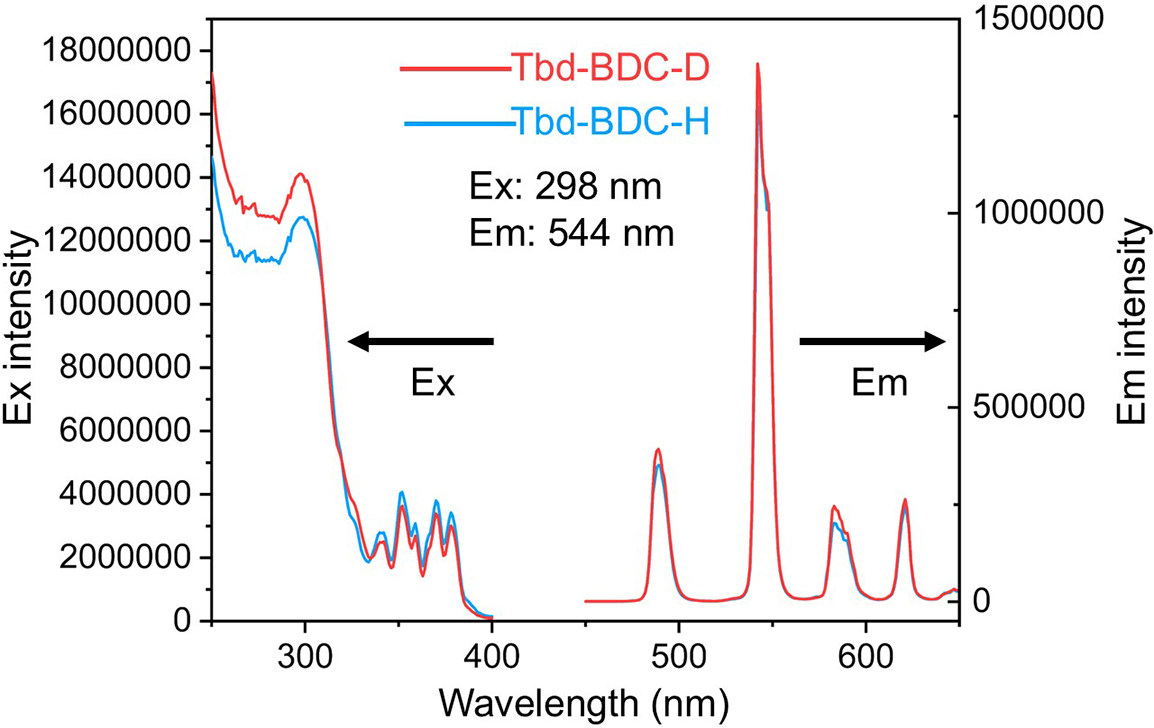


**Figure S48.** The photoluminescence (PL) excitation and emission spectra of Tbd-BDC-H/D.


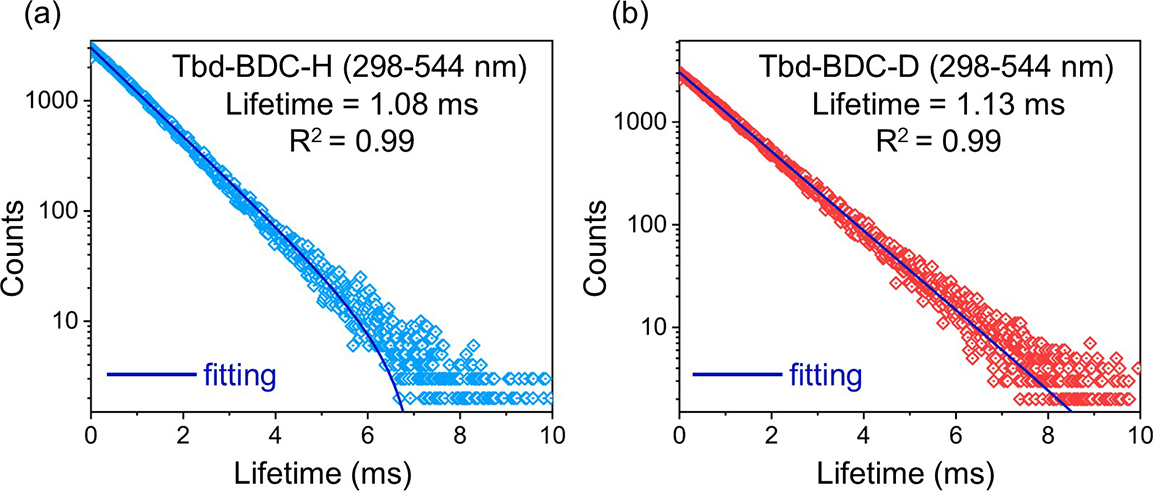


**Figure S49.** (a) Fitting of the lifetime of Tbd-BDC-H based on PL data (Ex/Em = 298/544 nm). (b) Fitting of the lifetime of Tbd-BDC-D based on PL data (Ex/Em = 298/544 nm).


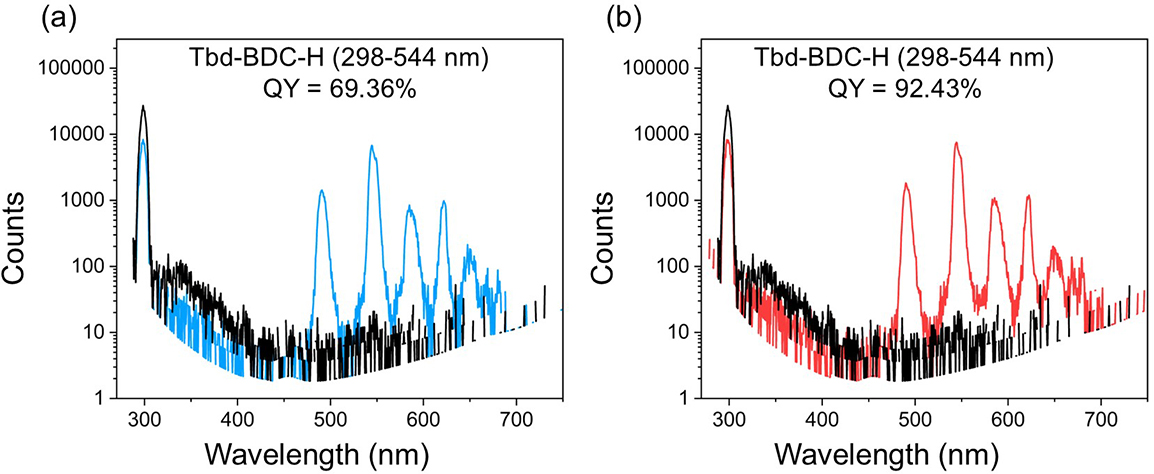


**Figure S50.** (a) Quantum yield of Tbd-BDC-H based on PL data (Ex/Em = 298/544 nm, black line for background). (b) Quantum yield of Tbd-BDC-D based on PL data (Ex/Em = 298/544 nm, black line for background).


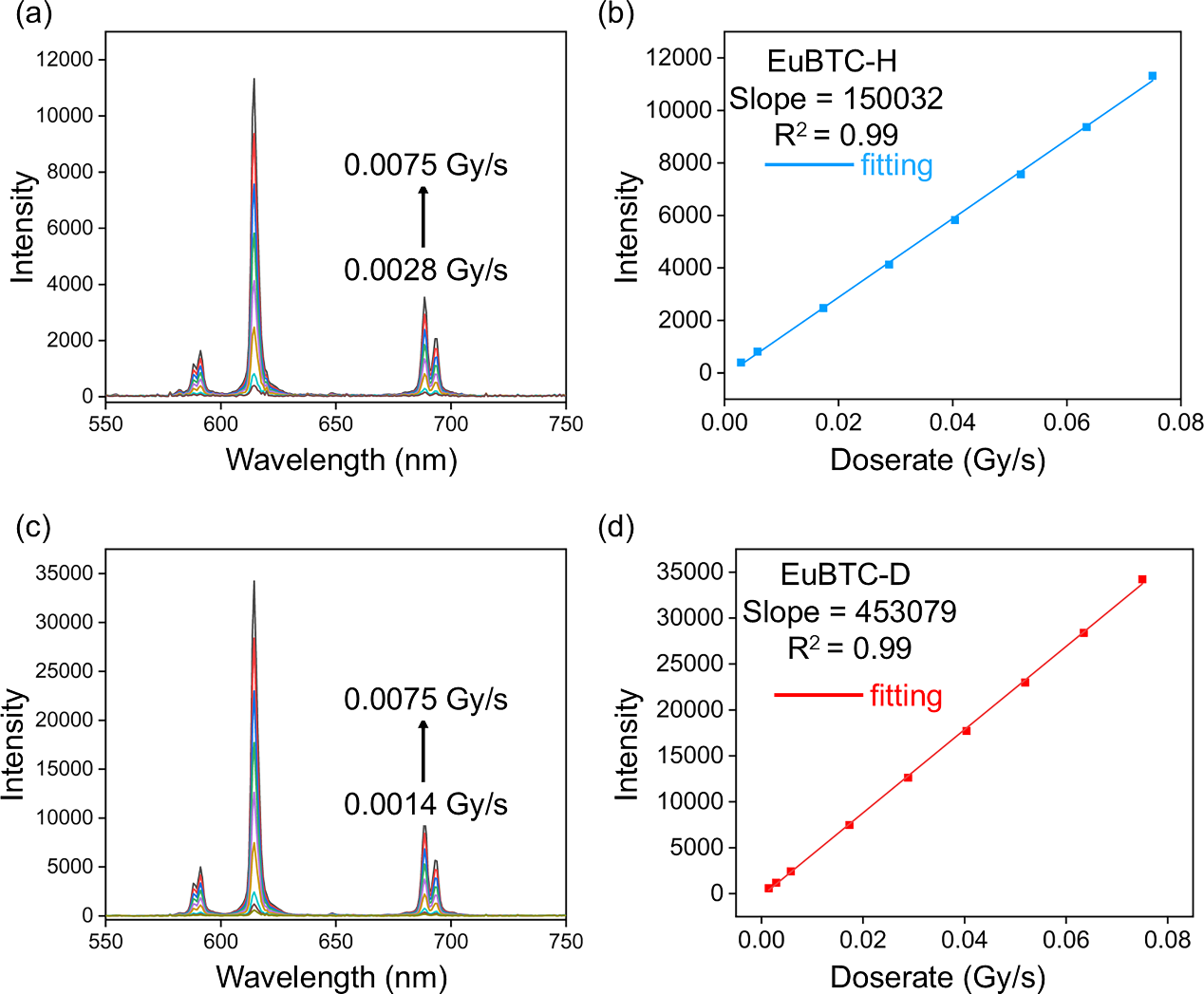


**Figure S51.** XEL spectra and linear fitting between X-ray dose rate and XEL intensity of EuBTC-H/D. (a) XEL spectra under different X-ray dose rate of EuBTC-H. (b) The linear fitting between X-ray dose rate and XEL intensity of EuBTC-H. (c) XEL spectra under different X-ray dose rate of EuBTC-D. (b) The linear fitting between X-ray dose rate and XEL intensity of EuBTC-D.


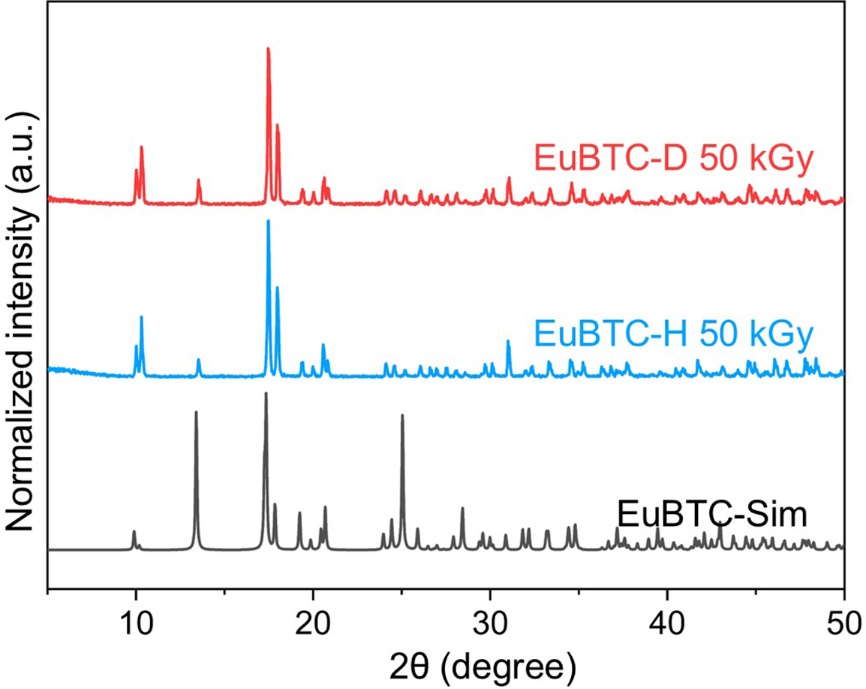


**Figure S52.** Comparison of PXRD patterns between EuBTC-H/D (under a continued γ-ray expose with the dose of 50 kGy) and simulation of single crystal.


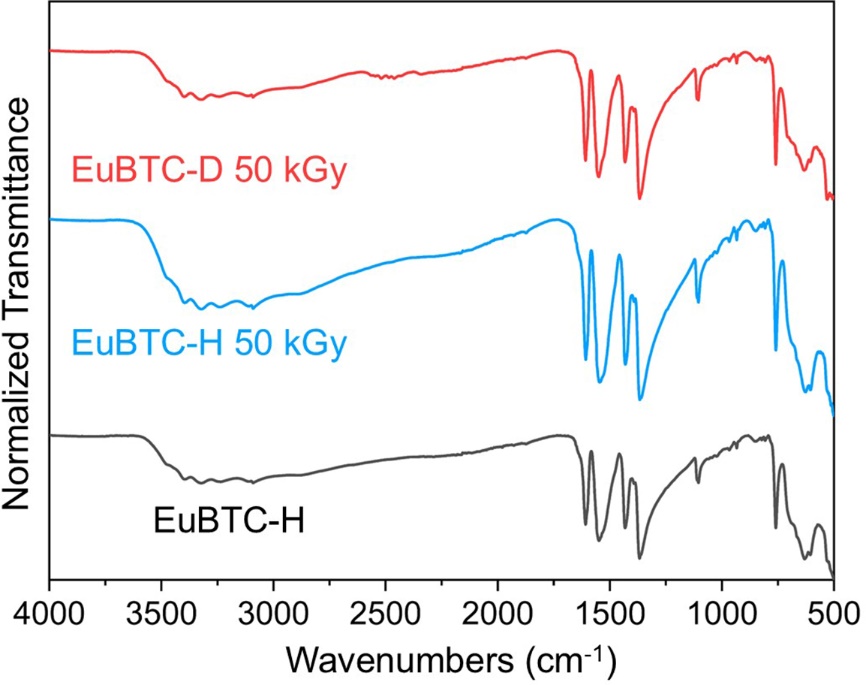


**Figure S53.** Comparison of FT-IR spectrum between between EuBTC-H/D (under a continued γ-ray expose with the dose of 50 kGy)


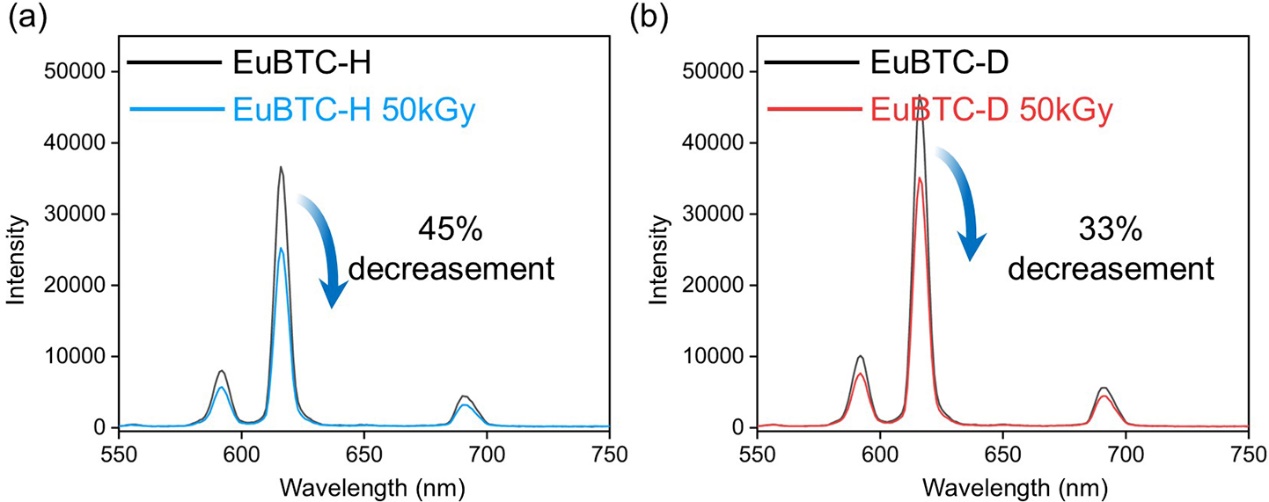
**Figure S54.** (a) The XEL spectra of EuBTC-H and EuBTC-H-50-kGy (the counterpart under a continued γ-ray expose with the dose of 50 kGy). (b) The XEL spectra of EuBTC-D and EuBTC-D-50-kGy (the counterpart under a continued γ-ray expose with the dose of 50 kGy).


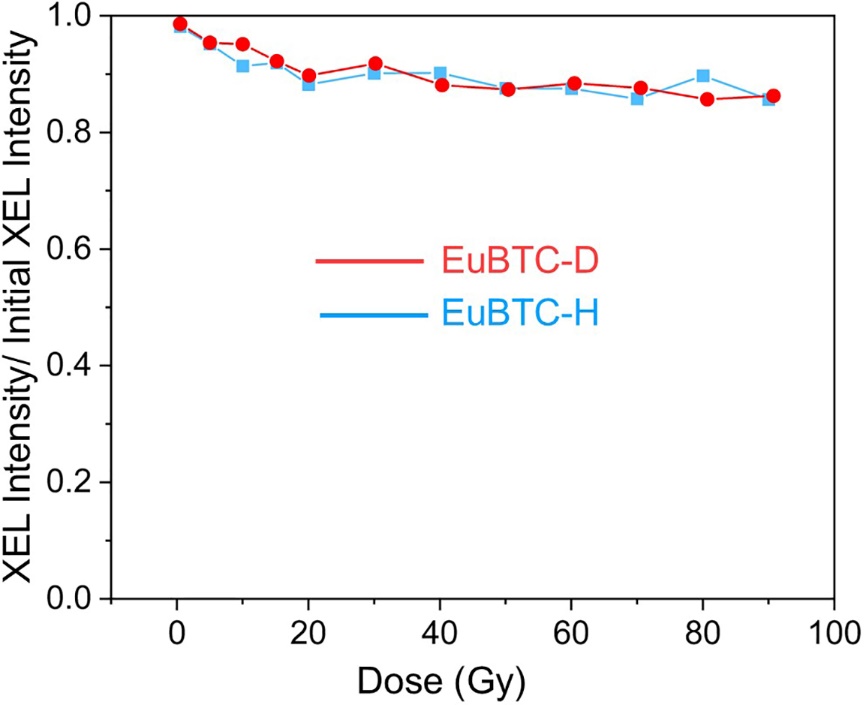


**Figure S55.** Normalized comparison of the initial XEL intensity of EuBTC-H/D with increasing X-ray irradiation dose of 90 Gy.


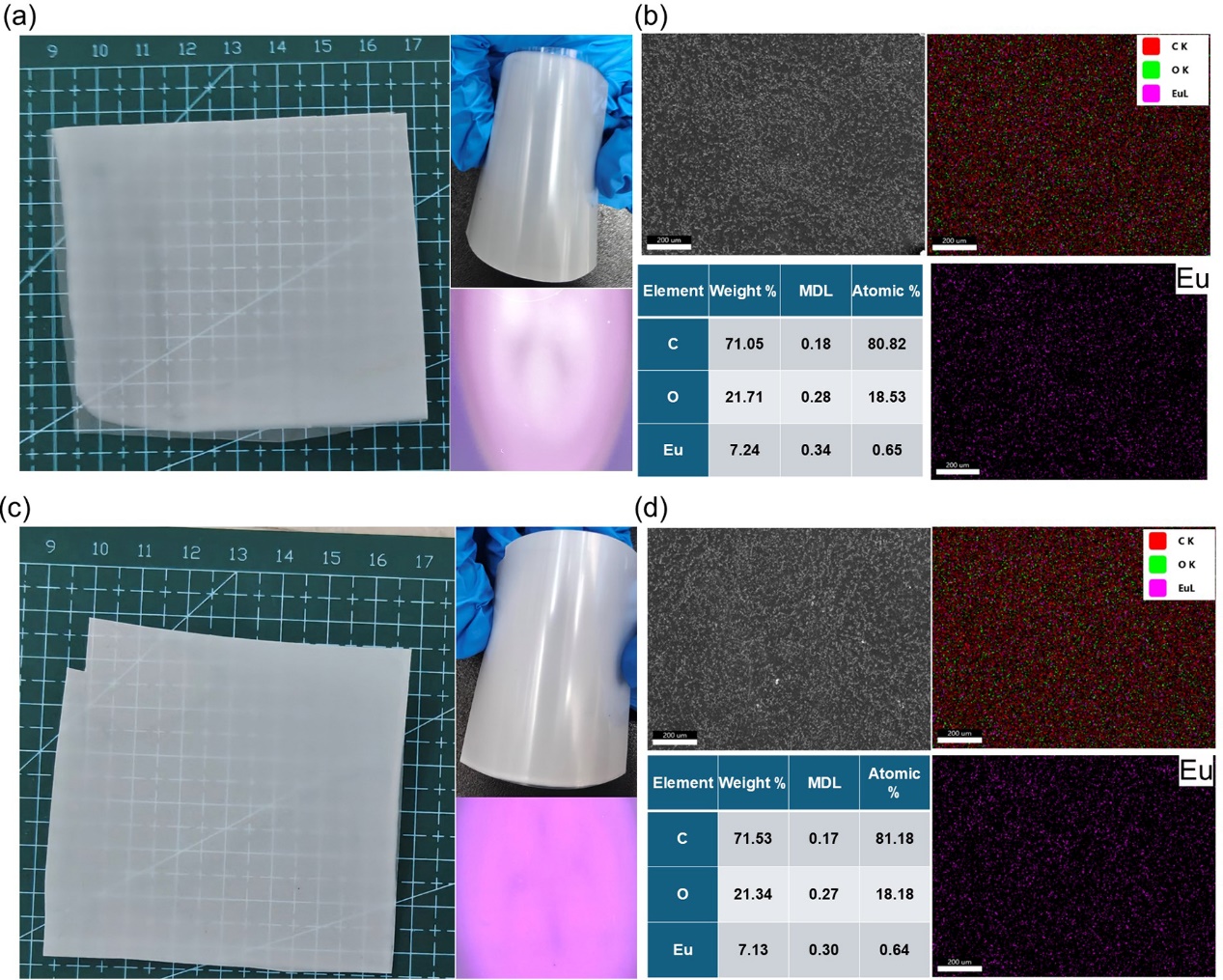


**Figure S56.** The photograph of the flexible film based on (a) EuBTC-H and (c) EuBTC-D prepared for imaging devices and the corresponding SEM-mapping images of (b) EuBTC-H and (d) EuBTC-D.


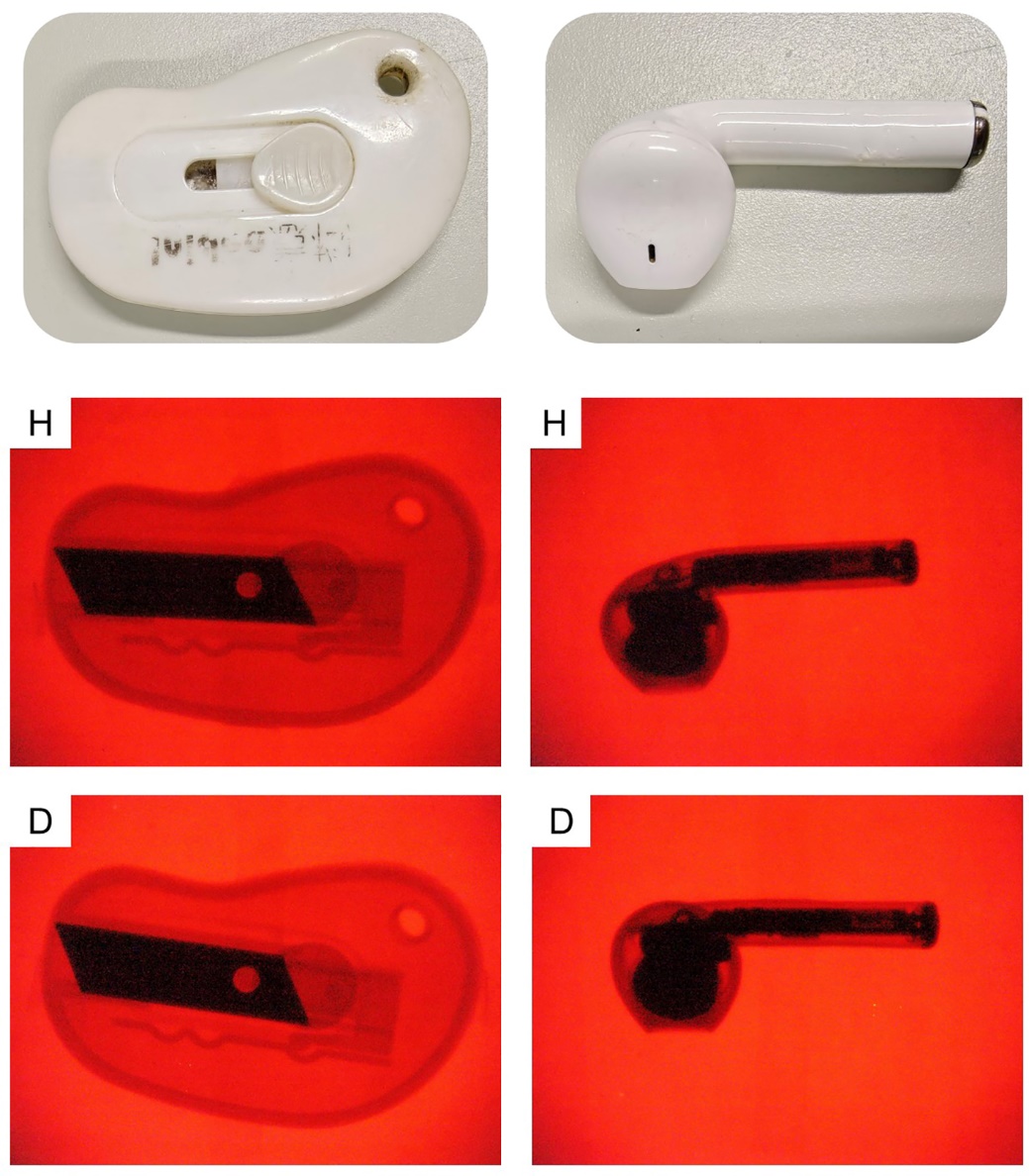


**Figure S57.** The original X-ray images of the two objects.


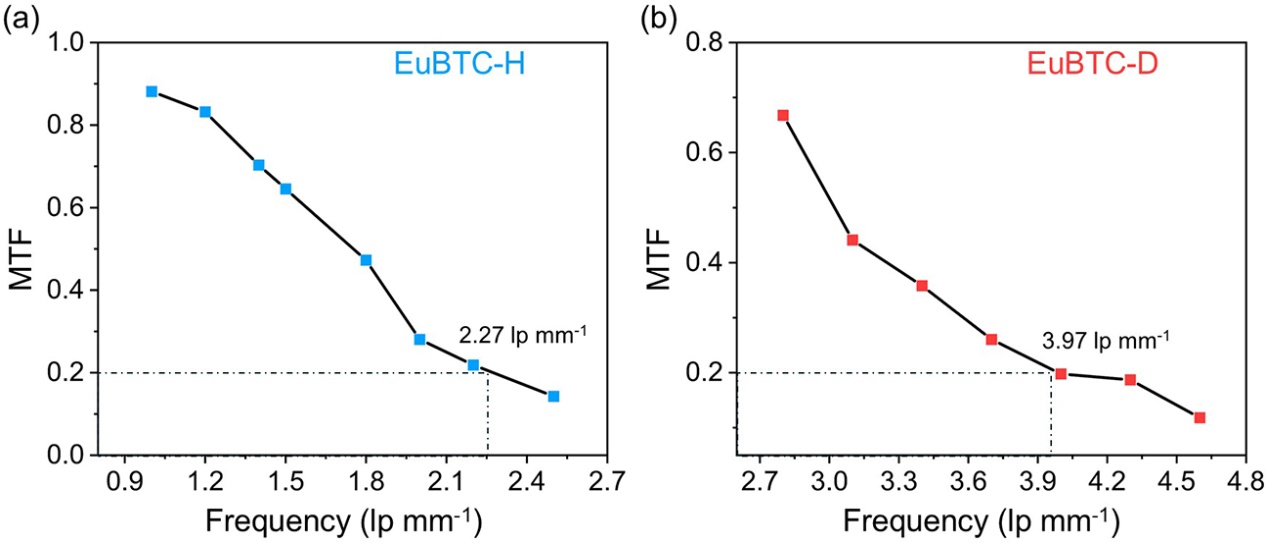


**Figure S58.** MTF curves of the scintillator membranes of (a) EuBTC-H and (b) EuBTC-D.


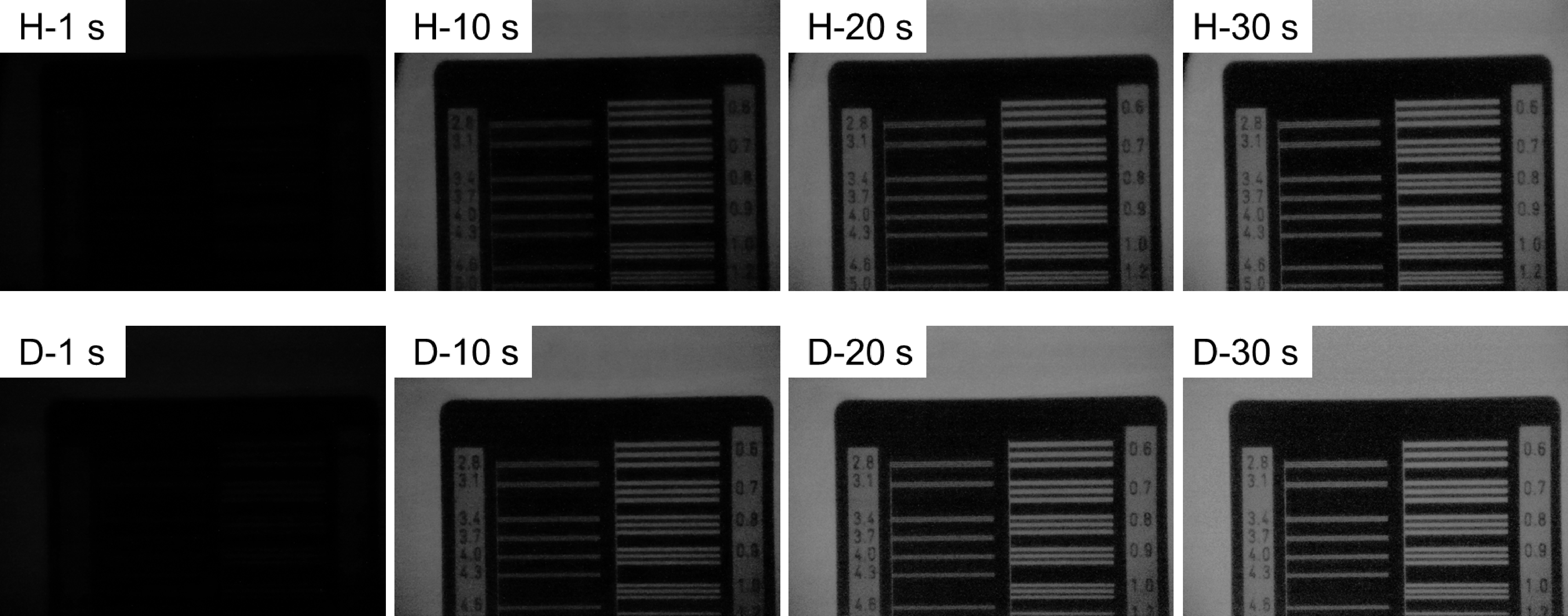


**Figure S59.** X-ray images conducted with the flexible films based on EuBTC-H/D powders with different exposure time.

**Section S2. Supplementary table**

**Table S1. The deuterium abundance of EuBTC-H/D and EuBTC-D-post.**

|  | EuBTC-H | EuBTC-D | EuBTC-D-post |
| --- | --- | --- | --- |
| H/% | 99.9837 | 83.73 | 99.8448 |
| D/% | 0.0163 | 16.27 | 0.1552 |
| D/H | 0.000163 | 0.194342 | 0.001554 |

**Table S2. The LT, QY, k_r/non_, and structure information of all as-synthesized MOFs. (coordinated water molecules-CWM)**

|  | **LT** | **LT-D/LT-H** | **QY** | **QY-D/QY-H** | **CWM** | **Structure** | **k_r_/s** | **k_r_/k_total_** | **k_non_** | **k_non(H/D)_** | **k_non_/k_total_** |
| --- | --- | --- | --- | --- | --- | --- | --- | --- | --- | --- | --- |
| **EuBTC-H** | **1.94x10^-4^** | **5.15** | **0.0407** | **4.49** | **6** | **rigid** | **2.10×10^2^** | **0.041** | **4.94×10^3^** | **6.05** | **0.959** |
| **EuBTC-D** | **1.00x10^-3^** |  | **0.1829** |  |  |  | **1.82×10^2^** | **0.182** | **8.17×10^2^** |  | **0.818** |
| **TbBTC-H** | **5.54x10^-4^** | **1.8** | **0.315** | **1.53** | **6** | **rigid** | **5.69×10^2^** | **0.31** | **1.24×10^3^** | **2.39** | **0.69** |
| **TbBTC-D** | **1.00x10^-3^** |  | **0.4812** |  |  |  | **4.81×10^2^** | **0.48** | **5.19×10^2^** |  | **0.52** |
| **EuBDC-H** | **9.40x10^-4^** | **1.12** | **0.5427** | **1.05** | **1** | **rigid** | **5.77×10^2^** | **0.54** | **4.83×10^2^** | **1.18** | **0.46** |
| **EuBDC-D** | **1.05x10^-3^** |  | **0.5679** |  |  |  | **5.41×10^2^** | **0.57** | **4.11×10^2^** |  | **0.43** |
| **TbBDC-H** | **9.40×10^-4^** | **1.03** | **0.4102** | **2.03** | **1** | **rigid** | **4.36×10^2^** | **0.41** | **6.27×10^2^** | **3.60** | **0.59** |
| **TbBDC-D** | **9.68×10^-4^** |  | **0.8320** |  |  |  | **8.59×10^2^** | **0.83** | **1.74×10^2^** |  | **0.17** |
| **BTECTb-H** | **1.02×10^-3^** | **1.19** | **0.4428** | **1.38** | **1** | **rigid** | **4.34×10^2^** | **0.44** | **5.46×10^2^** | **1.69** | **0.56** |
| **BTECTb -D** | **1.21×10^-3^** |  | **0.6092** |  |  |  | **5.03×10^2^** | **0.61** | **3.23×10^2^** |  | **0.39** |
| **DMTPEu-H** | **1.51×10^-4^** | **1.19** | **0.0192** | **1.12** | **3** | **flexible** | **1.27×10^2^** | **0.019** | **6.50×10^3^** | **1.19** | **0.981** |
| **DMTPEu-D** | **1.80×10^-4^** |  | **0.0215** |  |  |  | **1.19×10^2^** | **0.021** | **5.44×10^3^** |  | **0.979** |

**Table S3. The LT, QY, k_r/non_, and deuteration information of all solid materials.**

|  | **LT/s** | **LT-D/LT-H** | **QY** | **QY-D/QY-H** | **material** | **method** | **k_r_/s** | **k_r_/k_total_** | **k_non_** | **k_non(H/D)_** | **k_non_/k_total_** | **Ref** |
| --- | --- | --- | --- | --- | --- | --- | --- | --- | --- | --- | --- | --- |
| **DR** | **3.0x10^-8^** | **1.73** | **0.133** | **1.71** | **OLED** | **C-H/D** | **4.4×10^6^** | **0.13** | **2.9×10^7^** | **1.93** | **0.87** | **^[1]^** |
| **D-DR** | **5.2x10^-8^** |  | **0.228** |  |  |  | **4.4×10^6^** | **0.23** | **1.5×10^7^** |  | **0.77** |  |
| **tz1/CPB** | **-** | **-** | **0.20** | **1.46** | **OLED** | **C-H/D** | **-** | **-** | **-** |  | **-** | **^[2]^** |
| **tz1-d/CPB-d** | **-** |  | **0.122** |  |  |  | **-** | **-** | **-** |  | **-** |  |
| **BBTTPA:mCP** | **2.0x10^-9^** | **2.35** | **0.05** | **3** | **OLED** | **C-H/D** | **2.5×10^7^** | **0.33** | **5.0×10^7^** | **2.38** | **0.67** | **^[3]^** |
| **(BBTTPA:mCP)-D** | **4.7x10^-9^** |  | **0.15** |  |  |  | **3.2×10^7^** | **0.60** | **2.1×10^7^** |  | **0.40** |  |
| **5CzBN** | **-** | **-** | **0.75** | **1.17** | **OLED** | **C-H/D** | **-** | **-** | **-** |  | **-** | **^[4]^** |
| **D-5CzBN** | **-** |  | **0.88** |  |  |  | **-** | **-** | **-** |  | **-** |  |
| **H-3-f** | **2.9x10^-7^** | **1.86** | **0.285** | **1.74** | **OLED** | **C-H/D** | **9.8×10^5^** | **0.28** | **2.47×10^6^** | **2.66** | **0.72** | **^[5]^** |
| **D-3-f** | **5.4x10^-7^** |  | **0.496** |  |  |  | **9.2×10^5^** | **0.50** | **9.3×10^5^** |  | **0.50** |  |
| **HPh-3-f** | **4.9x10^-7^** | **1.39** | **0.500** | **1.34** | **OLED** | **C-H/D** | **1.02×10^6^** | **0.50** | **1.02×10^6^** | **2.08** | **0.50** | **^[5]^** |
| **DPh-3-f** | **6.8x10^-7^** |  | **0.668** |  |  |  | **9.8×10^5^** | **0.67** | **4.9×10^5^** |  | **0.33** |  |
| **Dy(DPPOP)_3_** | **6.7x10^-5^** | **1.69** | **0.13** | **1.92** | **complexe** | **C-H/D** | **1.9×10^3^** | **0.13** | **1.3×10^4^** |  | **0.87** | **^[6]^** |
| **Dy(D-DPPOP)_3_** | **1.13x10^-4^** |  | **0.25** |  |  |  | **2.2x10^3^** | **0.25** | **6.6x10^3^** |  | **0.75** |  |
| **SCU-COF-9a** | **1.05x10^-9^** | **1.32** | **0.0046** | **19** | **COF** | **C-H/D** | **4.4×10^6^** | **0.0046** | **9.5×10^8^** | **1.44** | **0.9954** | **^[7]^** |
| **SCU-COF-9d** | **1.39x10^-9^** |  | **0.0795** |  |  |  | **5.7×10^7^** | **0.0086** | **6.6×10^8^** |  | **0.9914** |  |
| **HH-COF** | **2.60x10^-9^** | **1.07** | **0.43** | **1.88** | **COF** | **C-H/D** | **1.7×10^8^** | **0.44** | **2.2×10^8^** | **3.14** | **0.56** | **^[8]^** |
| **DD-COF** | **2.78x10^-9^** |  | **0.81** |  |  |  | **2.9×10^8^** | **0.81** | **7×10^7^** |  | **0.19** |  |
| **HHOF** | **-** | **-** | **0.62** | **1.11** | **HOF** | **N-H/D** | **-** | **-** | **-** |  | **-** | **^[9]^** |
| **DHOF** | **-** |  | **0.69** |  |  |  | **-** | **-** | **-** |  | **-** |  |
| **BdCH_3_/CzCH_3_** | **0.388** | **4.82** | **0.023** | **1.26** | **RTP** | **C-H/D** | **0.17** | **0.066** | **2.41** | **4.73** | **0.934** | **^[10]^** |
| **BdD8CD_3_/CzCH_3_** | **1.870** |  | **0.029** |  |  |  | **0.03** | **0.056** | **0.51** |  | **0.944** |  |
| **Bd/Cz** | **0.485** | **3.65** | **0.009** | **1.44** | **RTP** | **C-H/D** | **0.05** | **0.024** | **2.01** | **3.65** | **0.976** | **^[10]^** |
| **BdD8/Cz** | **1.771** |  | **0.013** |  |  |  | **0.02** | **0.035** | **0.55** |  | **0.965** |  |
| **TPE** | **3.62 x10^-9^** | **1.17** | **0.211** | **1.16** | **molecule** | **C-H/D** | **5.83×10^7^** | **0.21** | **21.79×10^7^** | **1.11** | **0.79** | **^[11]^** |
| **TPE-20d** | **4.22x10^-9^** |  | **0.244** |  |  |  | **5.78×10^7^** | **0.23** | **19.71×10^7^** |  | **0.77** |  |
| **EuBTC-H** | **1.94x10^-4^** | **5.15** | **0.0407** | **4.49** | **Ln-MOF** | **O-H/D** | **2.10×10^2^** | **0.041** | **4.94×10^3^** | **6.05** | **0.959** | **This work** |
| **EuBTC-D** | **1.00x10^-3^** |  | **0.1829** |  |  |  | **1.82×10^2^** | **0.182** | **8.17×10^2^** |  | **0.818** |  |

**Table S4. The LT information of Eu^3+^** **in different solution environments.**

|  | **solvent** | **LT/s** | **LT-D/LT-H** | **QY** | **QY-D/QY-H** | **Ref** |
| --- | --- | --- | --- | --- | --- | --- |
| **Eu^3+^** | **H_2_O** | **1.101×10^-4^** | **33.6** |  |  | **^[12]^** |
| **Eu^3+^** | **D_2_O** | **3.703×10^-3^** |  |  |  |  |
| **Eu^3+^** | **H_2_O/DMF** | **2.52×10^-4^** | **5.6** |  |  | **^[12]^** |
| **Eu^3+^** | **D_2_O/DMF** | **1.40×10^-3^** |  |  |  |  |
| **Eu^3+^ + BDC** | **H_2_O/DMF** | **2.734×10^-4^** | **6.3** |  |  | **^[12]^** |
| **Eu^3+^ + BDC** | **D_2_O/DMF** | **1.724×10^-3^** |  |  |  |  |
| **EuBDC-H** | **-** | **9.40×10^-4^** | **1.12** |  |  | **[This work]** |
| **EuBDC-D** | **-** | **1.05×10^-3^** |  |  |  |  |
| **Eu^3+^** | **H_2_O** | **-** | **-** | **0.02** | **13** | **^[13]^** |
| **Eu^3+^** | **D_2_O** | **-** |  | **0.26** |  |  |
| **Eu^3+^** | **DMF** | **-** | **-** | **0.4** | **2** | **^[13]^** |
| **Eu^3+^** | **d_7_-DMF** | **-** |  | **0.8** |  |  |

**Table S5. The lifetime, quantum and synthesis of TbBDC-H/D and Tbd-BDC-H/D.**

|  | TbBDC-H | Tbd-BDC-H | TbBDC-D | Tbd-BDC-D |
| --- | --- | --- | --- | --- |
| Ligand | BDC | d-BDC | BDC | d-BDC |
| Solvent | DMF/H_2_O | DMF/H_2_O | DMF/D_2_O | DMF/D_2_O |
| Lifetime/ms | 0.94 | 1.08 | 0.97 | 1.13 |
| Quantum yield | 41.02 | 69.36 | 83.2 | 92.43 |

**Section S3. References**

[1] S. Wang, B. Su, X. Wang, Y. Wei, K. Kuo, C. Wang, S. Liu, L. Liao, W. Hung, L. Fu, W. Chuang, M. Qin, X. Lu, C. You, Y. Chi, P. Chou, *Nat. Photonics* **2022**, *16*, 843-850.

[2] X. Peng, C. H. Yeh, S. F. Wang, J. Yan, S. Gan, S. J. Su, X. Zhou, Y. X. Zhang, Y. Chi, *Adv. Opt. Mater* **2022**, *10*, 2201291.

[3] Q. Yu, Y. Tamura, H. Nakanotani, M. Mamada, C. Adachi, *Adv. Opt. Mater* **2024**, *12*, 2400932.

[4] T. Huang, Q. Wang, H. Zhang, Y. Zhang, G. Zhan, D. Zhang, L. Duan, *Nat. Photonics* **2024**, *18*, 516-523.

[5] S. Wang, D. Zhou, K. Kuo, C. Wang, C. Hung, J. Yan, L. Liao, W. Hung, Y. Chi, P. Chou, *Angew. Chem. Int. Ed.* **2024**, *63*, e202317571.

[6] Z. Cai, W. Yan, R. Guo, H. Liu, P. Huo, G. Yu, Z. Bian, Z. Liu, *Inorg. Chem.* **2023**, *62*, 6560-6564.

[7] M. Yuan, F. Ma, X. Dai, L. Chen, F. Zhai, L. He, M. Zhang, J. Chen, J. Shu, X. Wang, X. Wang, Y. Zhang, X. Fu, Z. Li, C. Guo, L. Chen, Z. Chai, S. Wang, *Angew. Chem. Int. Ed.* **2021**, *60*, 21250-21255.

[8] M. Yuan, F. Ma, L. Chen, B. Li, X. Dai, J. Shu, L. He, J. Chen, S. Lin, G. Xie, Z. Chai, S. Wang, *J. Am. Chem. Soc.* **2024**, *146*, 1250-1256.

[9] M. Yuan, F. Ma, Z. Jiang, L. Chen, Z. Chai, S. Wang, *Chem. Commun.* **2025**, *61*, 8755-8758.

[10] Z. Yin, Q. Sun, Z. Wu, Y. Xu, Z. Xie, B. Liu, *Aggregate* **2025**, *0*, e70141.

[11] S. Zhang, F. Ma, J. Jiang, B. Wu, J. Gong, G. Xu, C. Li, T. Chen, R. T. K. Kwok, J. W. Y. Lam, Z. Zhao, B. Z. Tang, *Angew. Chem. Int. Ed.* **2025**, *64*, e202511678.

[12] L. Chen, J. Lu, X. Li, N. Luan, Y. Song, S. Yang, M. Yuan, H. Qin, H. Zhu, X. Dong, K. Li, D. Zhang, L. Chen, X. Dai, Y. Wang, Y. Wang, C. Xu, Z. Chai, S. Wang, *J. Am. Chem. Soc.* **2024**, *146*, 6697-6705.

[13] Y. Wang, J. Wang, S. Zhang, N. Y. B. Tang, X. Ou, J. Jiang, F. Ma, P. Alam, Z. Qiu, W. Wang, Z. Zhao, J. W. Y. Lam, B. Z. Tang, *ACS Nano* **2025**, *19*, 25042-25051.
